# Effect of metformin on adverse outcomes in T2DM patients: Systemic review and meta-analysis of observational studies

**DOI:** 10.3389/fcvm.2022.944902

**Published:** 2022-09-23

**Authors:** Zhicheng Xu, Haidong Zhang, Chenghui Wu, Yuxiang Zheng, Jingzhou Jiang

**Affiliations:** ^1^Department of Cardiology, Jiangxi Provincial People's Hospital, The First Affiliated Hospital of Nanchang Medical College, Nanchang, China; ^2^Department of Nephrology, Peking University Third Hospital, Bejing, China; ^3^School of Medicine, Sun Yat-sen University, Shenzhen, China; ^4^Department of Anesthesiology, National Cancer Center/National Clinical Research Center for Cancer/Cancer Hospital, Chinese Academy of Medical Sciences and Peking Union Medical College, Beijing, China; ^5^Department of Cardiology, The First Affiliated Hospital, Sun Yat-sen University, Guangzhou, China

**Keywords:** metformin, type 2 diabetes mellitus, adverse outcomes, meta-analysis, cardiovascular

## Abstract

**Background:**

The cardiovascular protection effect of metformin on patients with type 2 diabetes mellitus (T2DM) remains inconclusive. This systemic review and meta-analysis were to estimate the effect of metformin on mortality and cardiovascular events among patients with T2DM.

**Methods:**

A search of the Pubmed and EMBASE databases up to December 2021 was performed. Adjusted hazard ratios (HRs) and 95% confidence intervals (CIs) were pooled by a random-effects model with an inverse variance method.

**Results:**

A total of 39 studies involving 2473009 T2DM patients were adopted. Compared to non-metformin therapy, the use of metformin was not significantly associated with a reduced risk of major adverse cardiovascular event (MACE) (HR = 1.06, 95%CI 0.91–1.22; *I*^2^ = 82%), hospitalization (HR = 0.85, 95%CI 0.64–1.13; *I*^2^ = 98%), heart failure (HR = 0.86, 95%CI 0.60–1.25; *I*^2^ = 99%), stroke (HR = 1.16, 95%CI 0.88–1.53; *I*^2^ = 84%), and risk of AMI (HR = 0.88, 95%CI 0.69–1.14; *I*^2^ = 88%) in T2DM patients. Metformin was also not associated with significantly lowered risk of MACE compared to dipeptidyl peptidase-4 inhibitor (DPP-4i) in T2DM patients (HR = 0.95, 95%CI 0.73–1.23; *I*^2^ = 84%).

**Conclusions:**

The effect of metformin on some cardiovascular outcomes was not significantly better than the non-metformin therapy or DPP-4i in T2DM patients based on observational studies.

## Introduction

Cardiovascular disease (CVD) is the predominant cause of death globally, resulting in a great economic burden and a tremendous threat to public health. Approximately 19.05 million deaths are estimated to CVD globally (1). Moreover, the incidence of CVD has been increasing or stagnating among younger individuals (aged 18–50 years) over the past few decades (2). Study shows that T2DM significantly increase the risk of CVD and aggressive glycemic control can reduce both macrovascular and microvascular events in T2DM patients (3).

Metformin, a biguanide derivative, has been used as a first-line hypoglycemic treatment for type 2 diabetes mellitus (T2DM) patients since 1957 when it was recommended by the European Association for the Study of Diabetes (EASD) and the American Diabetes Association (ADA) (4). Apart from its hypoglycemic effect, metformin has been found to confer protection against breast cancer (5), polycystic ovary syndrome (6), and neural recovery in patients with brain tumors (7). Metformin is also associated with a lower risk of major adverse cardiovascular events (MACEs) and all-cause mortality (8, 9). The UK Prospective Diabetes Study (UKPDS) shows that metformin has an effect of lowering the risk of cardiovascular morbidity and mortality over 20 years (10). Metformin can reduce the risk of heart failure, hospitalization, and stroke in patients with T2DM (10, 11). In T2DM patients with chronic kidney disease or heart failure, metformin may also show a cardiovascular protection effect (12, 13). Compared with other classic hypoglycemic agents (e.g., sulphonylurea), metformin reduces the risks of all-cause or cardiovascular mortality, stroke, and heart failure (14). When compared with new antidiabetic drugs such as sodium-glucose cotransporter-2 inhibitors, metformin is associated with a low rate of genital infection and ketoacidosis (15). However, recent conflicting reports have shown that metformin could not reduce all-cause and cardiovascular mortality (16, 17). Moreover, the combination of metformin and other hypoglycemic drugs may even impose higher death risks (17).

Han et al. (18) found Metformin could reduce cardiovascular mortality, all-cause mortality, and cardiovascular events in coronary artery disease (CAD) patients. However, Han's study only targeted CAD patients rather than the common population. Since more observational studies showed up and current observational evidence on the effect of metformin on CVD risk was still inconclusive, we carried out this meta-analysis on a synthesis of published data to estimate adverse cardiovascular outcomes following metformin treatment in patients with T2DM.

## Methods

Overall, the corresponding authors designed the research criteria, and two reviewers independently performed the literature search, study selection, data abstraction, quality assessment, and data analysis. Disagreements were resolved by discussion between two reviewers, or consultation with the corresponding authors. Ethical approval was not necessary for this study because only the published studies were included.

### Inclusion and exclusion criteria

We included observational retrospective or prospective cohort studies, in which the adverse outcomes were compared between patients with T2DM treated with metformin monotherapy and those treated with any other single drug or diet/lifestyle modification. The adverse outcomes of interest included all-cause mortality, MACE, hospitalization, heart failure, cardiovascular mortality, stroke, and AMI. The primary outcome was MACE, whereas others were secondary outcomes. The definitions of the studied outcomes were applied that were reported in the originally included studies.

We excluded studies focusing on patients with type 1 diabetes mellitus or patients without T2DM but with metformin treatment. Studies in which patients with T2DM were treated with two or more antidiabetic drugs or with one antidiabetic drug combined with insulin were also excluded. Certain publication types were excluded (e.g., reviews, comments, case reports, case series, letters, editorials, and meeting abstracts) due to insufficient data.

### Literature search

A prior meta-analysis by Han et al. (18) has studied the effect of metformin on adverse outcomes in patients with T2DM, and the end date of the literature search in this study was October 2019. Therefore, we abstracted the included studies for the meta-analysis by Han et al., and then systemically searched the PubMed and Embase databases from January 2019 to December 2021 to identify studies about the effect of metformin monotherapy vs. other treatments on adverse outcomes in patients with T2DM. The search terms combined with “AND” were applied as follows: (1) “metformin,” (2) “diabetes mellitus” OR “diabetes.” No linguistic restrictions were applied in the literature search.

### Study selection and data abstraction

We first screened the titles and abstracts of the retrieved studies from the PubMed and Embase databases, and subsequently, read the full texts of the potential studies. Those studies included in the prior meta-analysis by Han et al. (18) were also checked. Eligible studies would be chosen based on the pre-defined inclusion criteria. The following information of the included studies was collected: first author, publication year, country, study design, patient characteristics (study population, sample size, age, sex), follow-up time, type of treatment compared to metformin, sample size, and the number of events in the metformin and control groups, and adverse outcomes.

### Study quality assessment

The Newcastle-Ottawa Scale (NOS) tool was used to assess the quality of cohort studies. The NOS tool had three domains with a total of nine points: the selection of population (0–4 points), the comparability between experimental groups and control groups in the study (0–2 points), and the assessment of the outcome (0–3 points). In this meta-analysis, the study with a NOS score of less than 6 points was defined as low quality (19). This assessment method was used previously (20).

### Statistical analyzes

The statistical heterogeneity across the included studies was assessed using the *P*-value of the Cochrane Q test and the I^2^ statistic, where a *P* < 0.10 in the Cochrane Q test or an *I*^2^ > 50% suggested significant heterogeneity. The adjusted hazard ratios (HRs) and 95% confidence intervals (CIs) were considered as the effect sizes, and we converted them to the natural logarithms and standard errors, which were pooled by a random-effects model with an inverse variance method. The data analyses were performed based on the type of treatment and complications of patients. In the sensitivity analysis, we re-performed the above-mentioned analyses by deleting studies in which the sample size was smaller than 1,000 in either the control group or the experimental group. The publication bias for the reported effect estimates was assessed by funnel plots, egger and begg tests, and trim and fill analyses.

All the statistical analyses of this meta-analysis were performed using the Review Manager version 5.4 software (the Cochrane Collaboration 2014, Nordic Cochrane Centre Copenhagen, Denmark; https://community.cochrane.org/). In this study, a *P*-value of less than 0.05 was considered statistically significant.

## Results

### Study selection

The flow chart of the literature retrieval is presented in Supplementary Figure 1. A total of 10,244 studies were retrieved from the PubMed and Embase databases for the title and abstract screenings, after which 32 potential studies from databases were pooled with the other 34 studies included in the prior meta-analysis by Han et al. (18) to receive full-text screenings. Then we excluded 27 studies for the following reasons: (1) 21 studies did not focus on the adverse outcomes we set in the inclusion criteria (21–39); (2) 3 studies included patients without type 2 diabetes mellitus (40–42); (3) 1 ongoing study without outcomes (43); (4) 1 research investigated methodology of estimating the effect of metformin (44); (5) 1 study used metformin as the baseline drug in combination therapy (45). Finally, 39 studies [8 prospective cohort studies (15, 46–52) and 31 retrospective cohort studies (11, 13, 19, 26, 53–79)] were included in this meta-analysis.

### Baseline characteristics of the included studies

Table 1 shows the baseline characteristics of the included studies. Twenty-seven studies investigated the effect of metformin on all-cause mortality (13, 19, 46–53, 55–59, 61, 62, 64–66, 68, 72–74, 77–79), 16 studies investigated the effect of metformin on risk of MACE (26, 49, 52, 55, 60, 62, 64, 65, 69, 72, 74–79), 18 studies investigated the effect of metformin on risk of hospitalization (11, 13, 15, 47, 49, 50, 52, 56–60, 63, 67, 69, 71–73), 14 studies investigated the effect of metformin on risk of heart failure (11, 15, 49, 50, 52, 53, 56, 57, 59, 67–69, 71, 73), 14 studies investigated the effect of metformin on cardiovascular mortality (19, 26, 46, 47, 49, 50, 56, 59, 60, 62, 64, 71, 74, 75), 8 studies investigated the effect of metformin on risk of stroke (15, 26, 49, 57, 65, 68, 69, 74), and 6 studies investigated the effect of metformin on the risk of AMI (15, 26, 49, 54, 69, 74). Only 1 retrospective cohort study by Liu et al. (19) had a low quality with 4 points assessed by the NOS tool.

**Table 1:** Baseline characteristics of included studies.

**Author**	**Year**	**Country**	**Study design**	**Patient characteristics**	**Sample size**	**Gender male %**	**Age**	**Follow-up (y)**	**Comparison**	**NOS for quality assessment**
Scheller	2014	Denmark	Retrospective cohort	T2DM	84,756	52	59.0 (15.2)	4	metformin vs. DPP-4i	9
Morgan	2014	UK	Retrospective cohort	T2DM	5,208	36	66.6 (10.4)	2.9/3.1	metformin vs. sulphonylurea	9
Roumie	2012	USA	Retrospective cohort	T2DM	161,296	97	65 (57–74)	5	metformin vs. sulphonylurea	9
Roumie	2017	USA	Retrospective cohort	T2DM	131,972	97	66 (57–75)	7.5	metformin vs. sulphonylurea	9
Wang	2017	USA	Retrospective cohort	T2DM	41,204	All	74.6 (5.8)	9	metformin vs. non-metformin	7
Fung	2015	China	Retrospective cohort	T2DM	11,293	40	61.70 (10.75	5	metformin vs. diet	8
Liu	2016	USA	Retrospective cohort	T2DM	272,149	44	60.7	7.4	metformin vs. sulphonylurea/ insulin	4
Facila	2017	Spain	Prospective cohort	T2DM+HF	835	56	71 (10)	2.4	metformin vs. non-metformin	8
Shah	2010	USA	Retrospective cohort	T2DM+HF	131	79	56 (11)	2	metformin vs. non-metformin	7
Romero	2011	Spain	Prospective cohort	T2DM+HF	1,184	47	70.5 (7.0)	9	metformin vs. non-metformin	8
Roussel	2010	France	Retrospective cohort	T2DM	19,691	66	67.1 (9.3)	2	metformin vs. non-metformin	8
Schramm	2011	Denmark	Retrospective cohort	T2DM	110,374	51	52.5 (14.0)	9	metformin vs. sulphonylurea /insulin	8
Duncan	2007	USA	Retrospective cohort	T2DM	1,284	76	65 (58–72)	0 (in-hospital)	metformin vs. non-metformin	7
Johnson	2005	Canada	Retrospective cohort	T2DM	4,142	52	64.3 (12.4)	9	metformin vs. sulphonylurea	7
Evans	2006	UK	Prospective cohort	T2DM	7,967	51	60.2	5	metformin vs. sulphonylurea	9
Chen	2016	Canada	Retrospective cohort	T2DM	179,742	53	52.53 (10.07)	6	metformin vs. diet	6
Sillars	2010	Australia	Prospective cohort	T2DM	1,271	44	60.6 (11.9)	15	metformin vs. sulphonylurea /insulin/diet	7
Abualsuod	2015	USA	Retrospective cohort	T2DM+AMI	720	52	60.42 (13.36)	1	metformin vs. non-metformin	7
Retwiński	2018	Poland	Retrospective cohort	T2DM+HF	1,030	70	64.5 (10.5)	1	metformin vs. non-metformin	8
Pantalone	2009	USA	Retrospective cohort	T2DM	20,450	42	56.8 (13.9)	6	metformin vs. rosiglitazone/pioglitazone/sulphonylurea	7
Whitlock	2020	Canada	retrospective cohort	T2DM+CKD	21,996	51	54.7 (16.1)/61.8 (16.8)	1.4/1.1	metformin vs. sulphonylurea	8
Roumie	2019	America	Retrospective cohort	T2DM+CKD	96,725	98	70	1/1.2	metformin vs. sulphonylurea	9
Clegg	2021	America	Retrospective cohort	T2DM+CKD	3,490	61	68.33	NA	metformin vs. non-metformin	8
Ritsinger	2020	Sweden	Prospective cohort	T2DM+AMI	70,270	70	68 (11)	3.4	metformin vs. diet	7
Baksh	2020	America	Retrospective cohort	T2DM	445,701	53	51 (35–65)	341 days	metformin vs. DPP-4i/sulphonylurea	8
Jung	2021	Korea	Retrospective cohort	T2DM+AMI	35,348	68	64.6 (9.52)	4.3	metformin vs. non-metformin	7
Richardson	2021	America	Retrospective cohort	T2DM+CKD	96,741	97	70	1/1.2	metformin vs. sulphonylurea	8
Jong	2019	Taiwan, China	Prospective cohort	T2DM	1,157	72	64.4	1.5	metformin vs. non-metformin	7
He	2021	China	Retrospective cohort	T2DM	24,099	44	59.2 (15)	2	metformin vs. non-metformin	7
Chen	2020	Taiwan, China	Retrospective cohort	T2DM	41,020	56/63%	59.3 (12.9)/57.6 (13.0)	1.5/1.6	metformin vs. SGLT2i	8
Wang	2021	China	Retrospective cohort	T2DM+HF	372	52	71	4	metformin vs. non-metformin	8
Gu	2020	China	Retrospective cohort	T2DM	390	58/55%	68.1 (6.9)/68.9 (6.6)	6	metformin vs. non-metformin	7
Khan	2021	America	Retrospective cohort	T2DM+HF	5,852	48	75	1	metformin vs. non-metformin	7
Fralick	2021	America	Prospective cohort	T2DM	19,928	48	54	213 days / 147days	metformin vs. SGLT2i	7
Bromage	2019	England	Prospective cohort	T2DM+AMI	4,030	62/57%	71.3/76.1	343 days	metformin vs. non-metformin	8
Kim	2021	Korea	Retrospective cohort	T2DM+CKD	97,713	63/70%	66.0 (8.9)/66.3 (9.5)	5.3	metformin vs. non-metformin	9
Tseng	2021	Taiwan, China	Retrospective cohort	T2DM	195,064	54/53%	68.77/64.23	6	metformin vs. non-metformin	8
Tseng	2019	Taiwan, China	Retrospective cohort	T2DM	216,286	54/50%	59.17/65.81	NA	metformin vs. non-metformin	6

T2DM, Type 2 Diabetes Mellitus; HF, Heart Failure; AMI, Acute Myocardial Infarction; CKD, Chronic Kidney Disease; RCT, Randomized Controlled Trial; DPP-4i, Dipeptidyl Peptidase-4 inhibitor; SGLT2i, Sodium-Dependent Glucose Transporter 2 inhibitor; NOS, Newcastle-Ottawa Scale; NA, Not Available.

### Effect of metformin on MACE in T2DM patients

**Figure 1:**
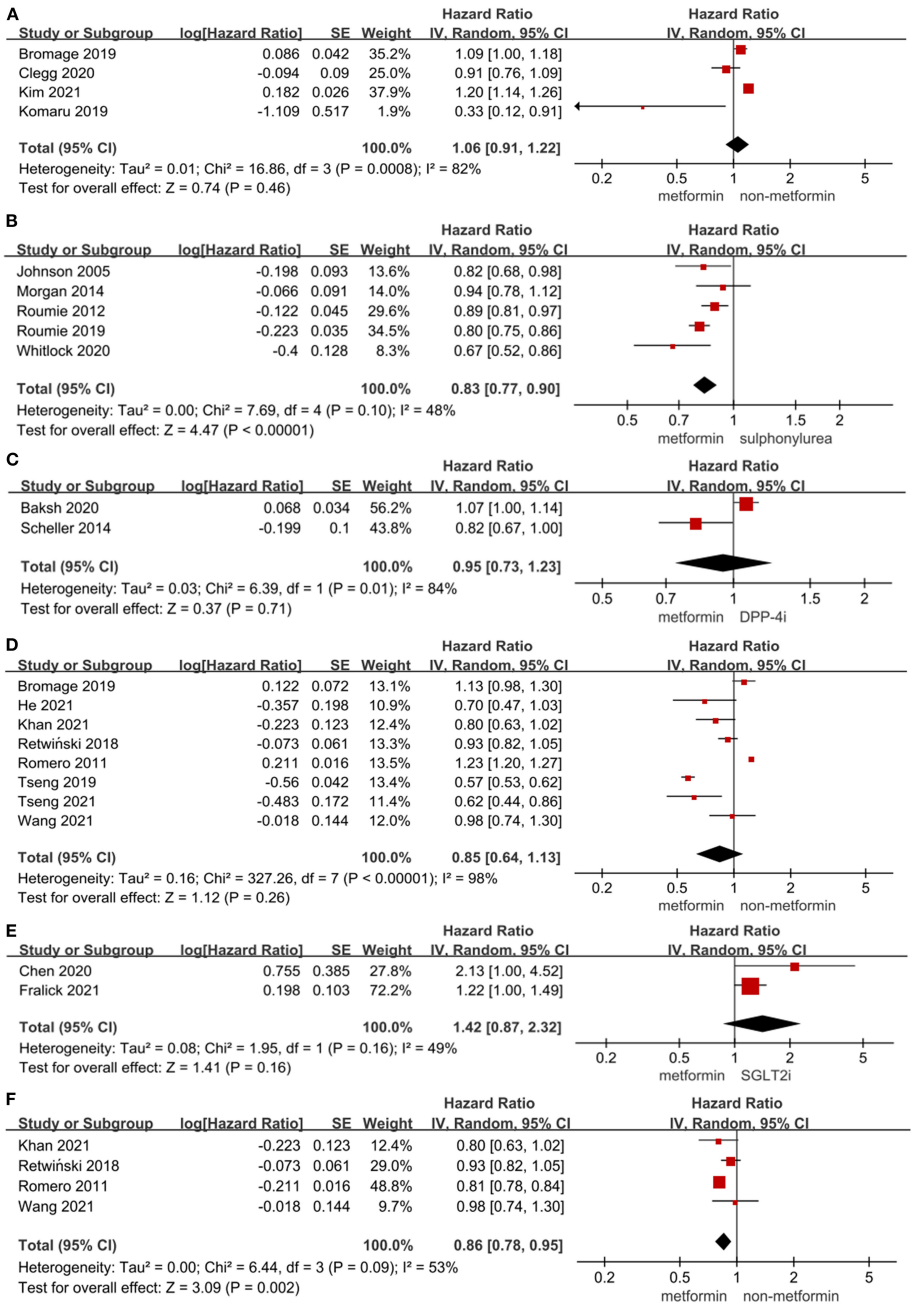
**(A)** Forest plot of hazard ratio of MACE among patients with metformin therapy vs non-metformin therapy. **(B)** Forest plot of hazard ratio of MACE among patients with metformin therapy vs. sulphonylurea therapy. **(C)** Forest plot of hazard ratio of MACE among patients with metformin therapy vs. DPP-4i therapy. **(D)** Forest plot of hazard ratio of hospitalization among patients with metformin therapy vs non-metformin therapy. **(E)** Forest plot of hazard ratio of hospitalization among patients with metformin therapy vs. SGLT2i. **(F)** Forest plot of hazard ratio of hospitalization among heart failure patients with metformin therapy vs non-metformin therapy. CI, confidence interval; SE, standard error; IV, inverse of the variance.

As shown in Figures 1A–C, the use of metformin was not associated with a decrease in the risk of MACE when compared to non-metformin (HR = 1.06, 95%CI 0.91–1.22; *I*^2^ = 82%). Specifically, metformin was associated with a decreased risk of MACE when compared to sulphonylurea (HR = 0.83, 95%CI 0.77–0.90; *I*^2^ = 48%). What's more, the use of metformin did not alter the risk of MACE significantly compared to dipeptidyl peptidase-4 inhibitor (DPP-4i) in T2DM patients (HR = 0.95, 95%CI 0.73–1.23; *I*^2^ = 84%).

### Effect of metformin on all-cause mortality in T2DM patients

**Figure 2:**
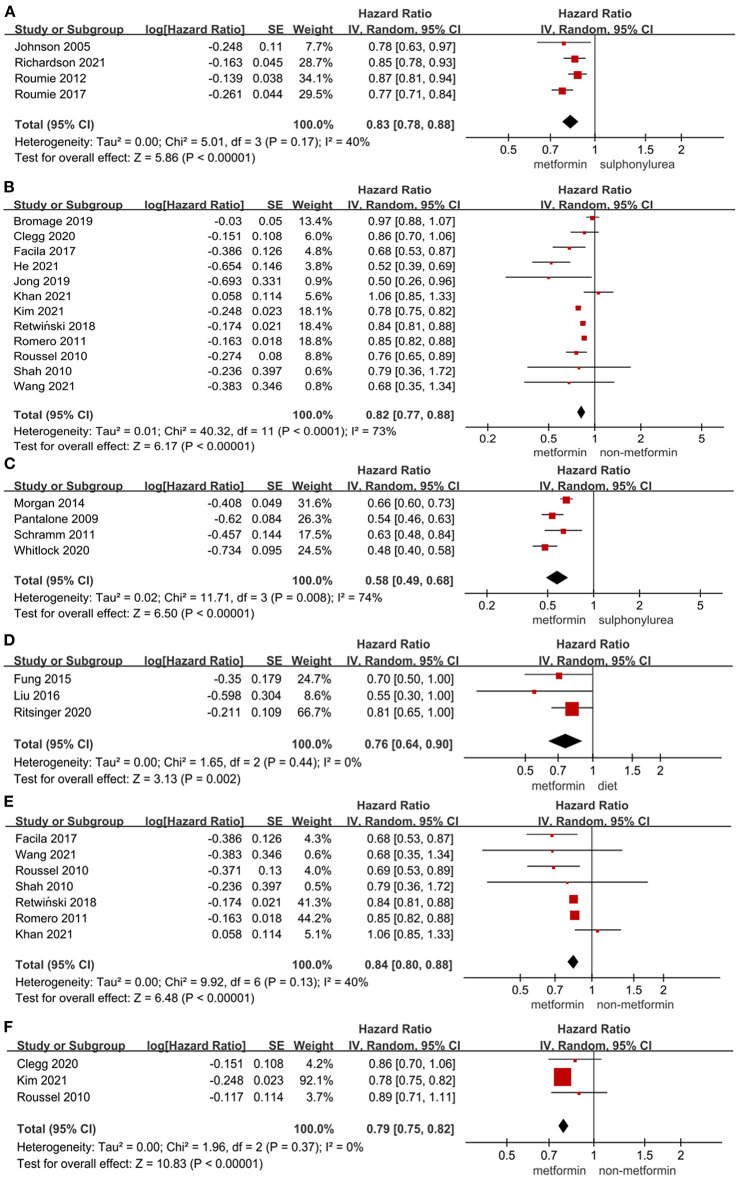
**(A)** Forest plot of hazard ratio of hospitalization among patients with metformin therapy vs. sulphonylurea therapy. **(B)** Forest plot of hazard ratio of all-cause mortality among patients with metformin therapy vs. non-metformin therapy. **(C)** Forest plot of hazard ratio of all-cause mortality among patients with metformin therapy vs. sulphonylurea therapy. **(D)** Forest plot of hazard ratio of all-cause mortality among patients with metformin vs. diet therapy. **(E)** Forest plot of hazard ratio of all-cause mortality among heart failure patients with metformin therapy vs. non-metformin therapy. **(F)** Forest plot of hazard ratio of all-cause mortality among patients with CKD treated with metformin therapy vs non-metformin therapy. CI, confidence interval; SE, standard error; IV, inverse of the variance.

The results of the effect of metformin on all-cause mortality in T2DM patients were presented in Figures 2B–F, showing that the use of metformin was associated with a significantly lower all-cause mortality in T2DM patients compared to non-metformin therapy (HR = 0.82, 95%CI 0.77–0.88; *I*^2^ = 73%), sulphonylurea (HR = 0.58, 95%CI 0.49–0.68; *I*^2^ = 74%), and diet therapy (HR = 0.76, 95%CI 0.64–0.90; *I*^2^ = 0%). Also, there was a significant reduction in all-cause mortality in T2DM patients with heart failure (HR = 0.84, 95%CI 0.84–0.88; *I*^2^ = 40%) or CKD (HR = 0.79, 95%CI 0.75–0.82; *I*^2^ = 0%) using metformin vs. non-metformin therapy. In addition, two studies by Scheller et al. (78) and Chen et al. (57), respectively, compared the effect of metformin on all-cause mortality in T2DM patients with DPP-4i (HR = 0.8, 95%CI 0.58–1.09) and sodium-dependent glucose transporter-2 inhibitor (SGLT-2i) (HR = 2.04, 95%CI 1.82–2.27).

### Effect of metformin on the risk of hospitalization in T2DM patients

Figures 1D–F, 2A presented the effect of metformin on hospitalization in T2DM patients. Metformin was not associated with a significant lower risk of hospitalization in T2DM patients compared to non-metformin therapy (HR = 0.85, 95%CI 0.64–1.13; *I*^2^ = 98%) and SGLT2i (HR = 1.42, 95%CI 0.87–2.32; *I*^2^ = 49%), but it significantly lowered the risk of hospitalization compared to non-metformin therapy (HR = 0.86, 95%CI 0.78–0.95; *I*^2^ = 53%) in T2DM patients with heart failure and sulphonylurea (HR = 0.83, 95%CI 0.78–0.88; *I*^2^ = 40%) in T2DM patients. And as reported by Baksh (69) the use of metformin was not associated with a significantly lower risk of hospitalization compared with DPP-4i in T2DM patients (HR = 1.04, 95%CI 0.77–1.40).

### Effect of metformin on the risk of heart failure in T2DM patients

**Figure 3:**
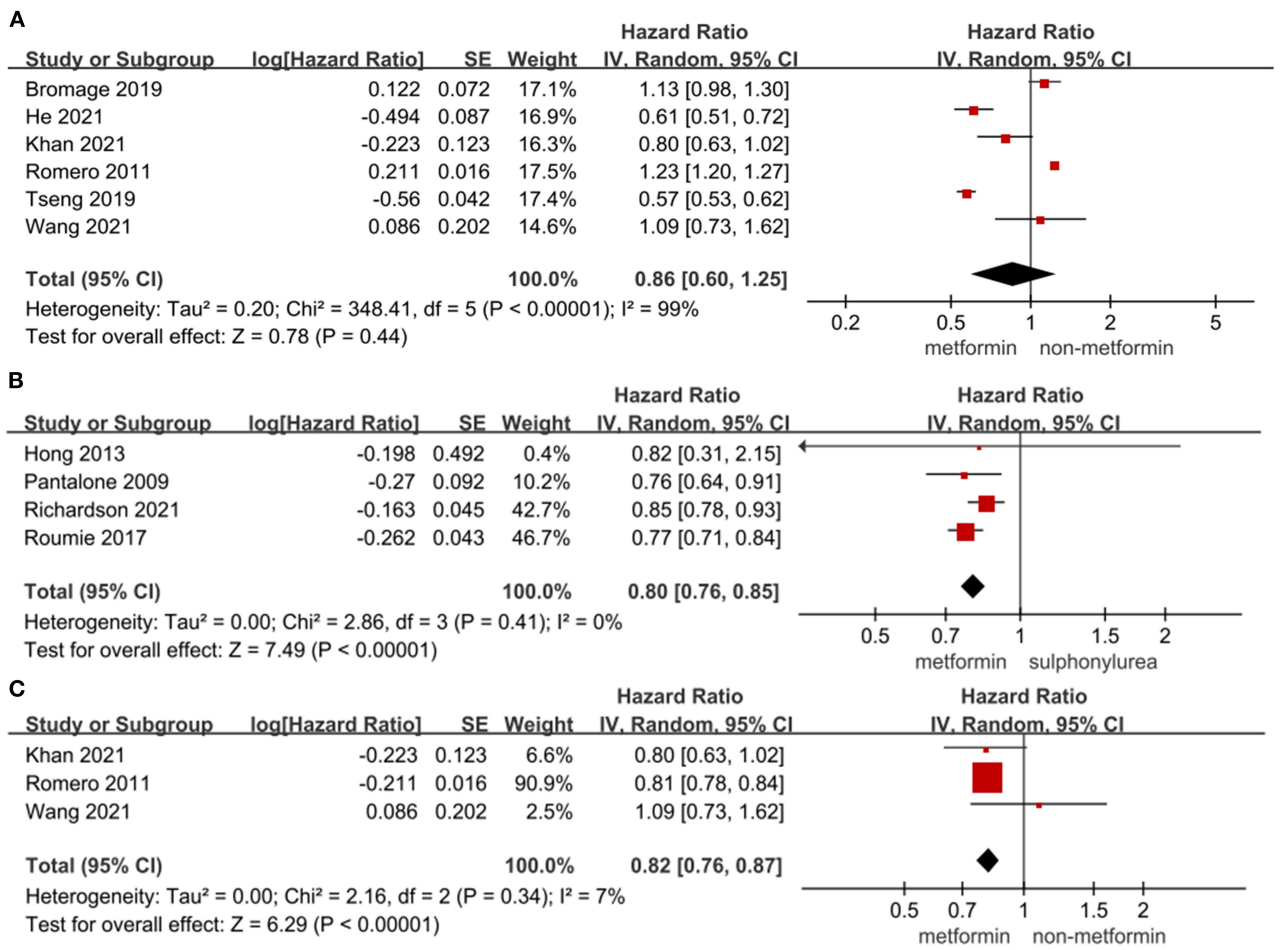
**(A)** Forest plot of hazard ratio of heart failure among patients with metformin therapy vs non-metformin therapy. **(B)** Forest plot of hazard ratio of heart failure among patients with metformin therapy vs. sulphonylurea therapy. **(C)** Forest plot of hazard ratio of the recurrent incident of heart failure among heart failure patients with metformin therapy vs. non-metformin therapy. CI, confidence interval; SE, standard error; IV, inverse of the variance.

As shown in Figure 3, the use of metformin was not associated with a significantly lower risk of heart failure in T2DM patients compared to non-metformin therapy (HR = 0.86, 95%CI 0.60–1.25; *I*^2^ = 99%). However, metformin significantly lowered the risk of heart failure compared to sulphonylurea (HR = 0.80, 95%CI 0.76–0.85; *I*^2^ = 0%) and the risk of recurrent incidents of heart failure compared to non-metformin therapy (HR = 0.82, 95%CI 0.76–0.87; *I*^2^ = 7%) in T2DM patients. And metformin was not significantly associated with reduced risk of heart failure compared to diet therapy (HR = 0.688, 95%CI 0.435–1.086) in T2DM patients in the study by Fung et al. (68) and compared to rosiglitazone (HR = 0.86, 95%CI 0.58–1.28) in T2DM patients in the study by Pantalone et al. (53).

### Effect of metformin on cardiovascular mortality in T2DM patients

**Figure 4:**
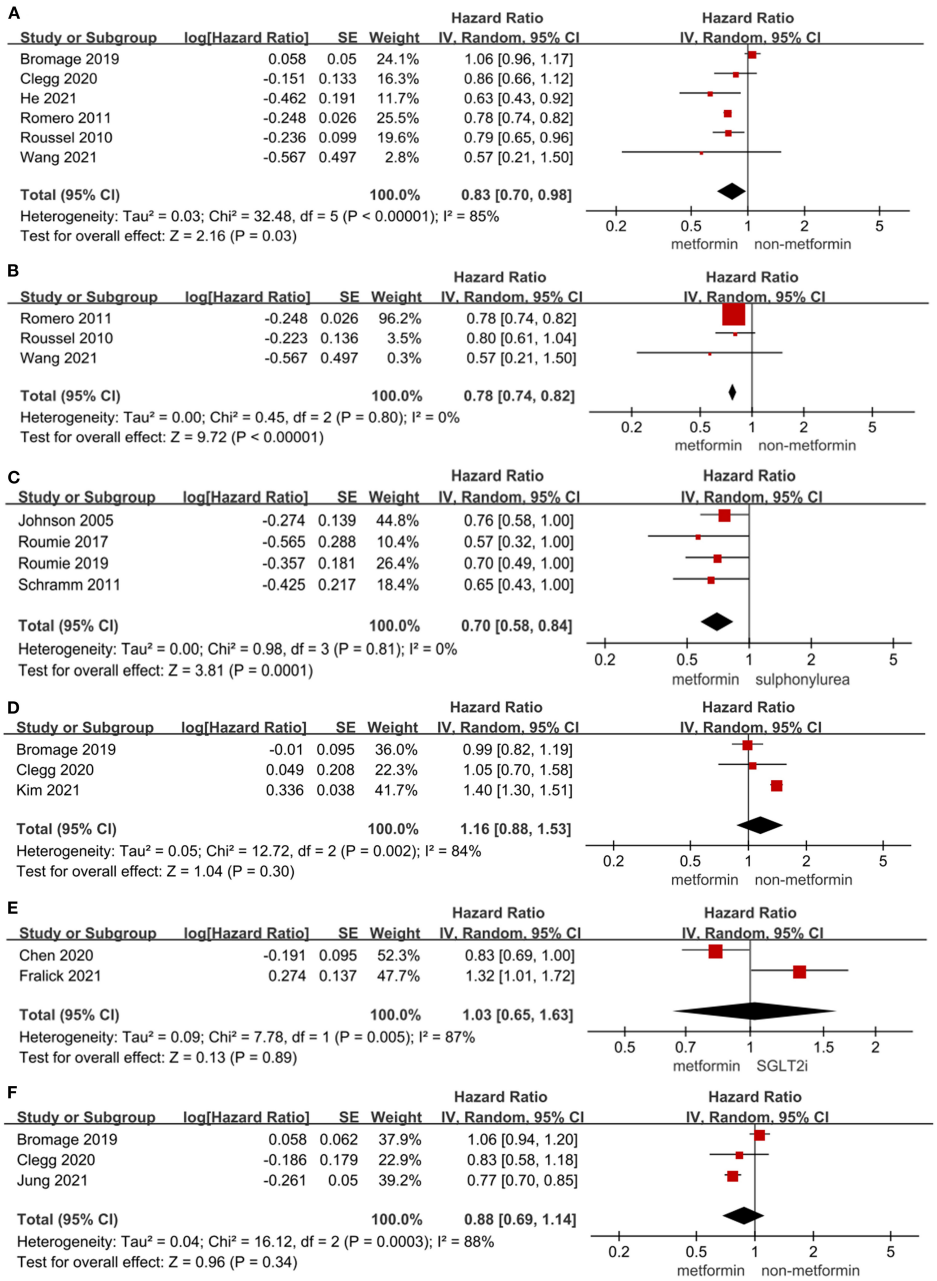
**(A)** Forest plot of hazard ratio of cardiovascular mortality among patients with metformin therapy vs non-metformin therapy. **(B)** Forest plot of hazard ratio of cardiovascular mortality among heart failure patients with metformin therapy vs. non-metformin therapy. **(C)** Forest plot of hazard ratio of cardiovascular mortality among patients with metformin therapy vs. sulphonylurea. **(D)** Forest plot of hazard ratio of stroke among patients with metformin therapy vs. non-metformin therapy. **(E)** Forest plot of hazard ratio of stroke among patients with metformin therapy vs. SGLT2i. **(F)** Forest plot of hazard ratio of AMI among patients with metformin therapy vs. non-metformin therapy. CI, confidence interval; SE, standard error; IV, inverse of the variance.

Figures 4A–C showed the effect of metformin on cardiovascular mortality in T2DM patients. The use of metformin was not only significantly associated with lower cardiovascular mortality in T2DM patients (HR = 0.83, 95%CI 0.70–0.98; *I*^2^ = 85%) and in T2DM patients with heart failure (HR = 0.78, 95%CI 0.74–0.82; *I*^2^ = 0%) compared to non-metformin therapy, but also significantly lowering cardiovascular mortality compared to sulphonylurea in T2DM patients (HR = 0.70, 95%CI 0.58–0.84; *I*^2^ = 0%). Liu et al. (19) reported that metformin vs. diet therapy was not associated with significantly lower cardiovascular mortality (HR = 0.87, 95%CI 0.45–1.68).

### Effect of metformin on the risk of stroke and AMI in T2DM patients

The effect of metformin on the risk of stroke and AMI in T2DM patients was shown in Figures 4D–F. The use of metformin was not associated with a significant decrease in the risk of stroke compared to non-metformin therapy (HR = 1.16, 95%CI 0.88–1.53; *I*^2^ = 84%) and SGLT2i (HR = 1.03, 95%CI 0.65–1.63; *I*^2^ = 87%) in T2DM patients. The use of metformin significantly lowered the risk of stroke compared to diet therapy in T2DM patients (HR = 0.698, 95%CI 0.511–0.954) in the study by Fung et al. (68) while the alteration of risk of stroke was not significant compared to DPP-4i (HR = 0.81, 95%CI 0.6–1.09) in T2DM patients in the study by Baksh et al. (69). And the risk of AMI did not decrease significantly in T2DM patients with metformin therapy vs. non-metformin therapy (HR = 0.88, 95%CI 0.69–1.14; *I*^2^ = 88%). What's more, the use of metformin was not associated with a significantly lower risk of AMI in T2DM patients compared to DPP-4i (HR = 0.95, 95%CI 0.72–1.27) and SGLT2i (HR = 1.10, 95%CI 0.66–1.85) in the study by Baksh et al. (69) and Fralick et al. (15), respectively.

### Sensitivity analysis and publication bias

Supplementary Table 2 showed the results of sensitivity analysis for the outcomes. The majority of the re-analyses showed similar results as the analysis before deleting studies with a sample size smaller than 1,000 in either the control group or the experimental group. However, only 1 study by Roussel et al. (64) remained after deleting the data in the analyses studying the effect of metformin on all-cause mortality and cardiovascular mortality vs. non-metformin therapy in T2DM patients with heart failure. And 0 study was left in the analyses studying the effect of metformin on the risk of hospitalization and the risk of heart failure vs. non-metformin therapy in T2DM patients with heart failure. Besides, the effect of metformin on cardiovascular mortality vs. non-metformin therapy in T2DM patients altered significantly (HR = 0.85, 95%CI 0.69–1.06; *I*^2^ = 78%) in the re-analysis compared to the one before deleting the data (HR = 0.83, 95%CI 0.70–0.98; *I*^2^ = 85%). This might mainly result from the long follow-up period (9 years) in the study by Romero et al. (50), suggesting that a longer follow-up period might better demonstrate the efficacy of metformin.

### Publication bias

The funnel plots and the results of egger and begg tests and trim and fill analyses in Supplementary Figures 2–22 indicated no potential publication biases for the adverse outcomes.

## Discussion

We included 39 subjects in this study involving 2473009 T2DM patients. We found:

(1) Metformin couldn't remarkably reduce the risk of MACEs compared to non-metformin therapy but could remarkably reduce the risk when compared with sulphonylurea in T2DM patients. (2) Metformin could significantly reduce cardiovascular mortality compared to non-metformin therapy in T2DM patients with or without heart failure or when compared with sulphonylurea in T2DM patients. (3) Metformin could significantly reduce all-cause mortality compared to non-metformin therapy, sulphonylurea, and diet therapy, and it could also significantly reduce all-cause mortality compared to non-metformin therapy in T2DM patients with heart failure or CKD. (4) Compared with non-metformin therapy, metformin was not effective in reducing the risk of heart failure in patients with T2DM, but it did reduce the risk of heart failure recurrence. Metformin was effective in reducing the risk of heart failure when compared with sulphonylurea in T2DM patients. (5) Metformin couldn't significantly reduce the risk of hospitalization compared to non-metformin therapy but could remarkably reduce the risk when compared with sulphonylurea in T2DM patients. Particularly, in patients with T2DM and heart failure, metformin can significantly reduce the risk of hospitalization. (6) Metformin couldn't remarkably reduce the risk of stroke compared to non-metformin therapy and SGLT2i in T2DM patients. (7) Metformin couldn't remarkably reduce the risk of AMI compared to non-metformin therapy in T2DM patients.

Major adverse cardiovascular events (MACEs) have different definitions in different studies. In this meta-analysis, some of the events were counted together as follows: AMI, stroke, heart failure, cardiovascular death, cardiac arrest, hospitalization, coronary angioplasty, transient ischemic attack, and unstable angina. T2DM is always related to cardiovascular complications. Since 1988, the study showed that in patients with T2DM, lowering blood glucose could reduce microvascular complications (80). There are usually exit raised levels of inflammatory cytokines among patients with T2DM. hyperglycemia and these inflammatory cytokines would harm the vascular endothelial cells, which would result in atherosclerosis in T2DM patients (81). At the same time, this condition decreases pro-angiogenic factors especially vascular endothelial growth factors and other collateral vessel growth-related parameters, which would impede collateral vessel growth (82). All of these are associated with CVD in T2DM patients to a great extent. Metformin has been thought to be protective of the cardiovascular system in the human body and here are some possible mechanisms: (I) Metformin was found to decrease cardiovascular inflammation and/or oxidative stress through activation of (AMP sensitive protein kinase) AMPK phosphorylation (83). (II)Metformin would attenuate atherosclerosis by the Inhibition of Drp1-mediated mitochondrial fission (84). However, in this meta-analysis, we found that metformin could not significantly reduce the risk of MACEs in T2DM patients. It might indicate that SGLT2i and DPP-4i have relatively the same cardiovascular protection ability as metformin. Considering the cardiovascular protection mechanism we mentioned above, it seemed to be unreasonable. And these results are opposite to some previous studies. A meta-analysis of randomized controlled trials found that metformin is significantly associated with lower risks of MACEs compared to placebo or other anti-hyperglycemic drugs among T2DM patients (85). A retrospective cohort analysis in China showed that metformin monotherapy could reduce the risk of heart failure in T2DM patients when compared with no metformin medications (59). A recent retrospective cohort analysis in Korea showed that metformin would significantly decrease the risk of AMI in all patients (54). But there also exist some studies showing the same results. Chang-Qian Wang found that metformin wasn't associated with a reduced risk of MACEs (76). A meta-analysis of 13 randomized trials showed that metformin couldn't remarkably reduce AMI and stroke (86), so there remains uncertainty about whether metformin reduces the risk of MACEs, AMI, stroke, and heart failure in patients with T2DM or not, and our included studies were limited (3 AMI studies, 3 stroke studies, and 4 MACEs studies) in this meta-analysis. More studies need to be implemented to test them.

All-cause mortality and cardiovascular mortality, which are vital living indicators in CVD patients, were found to be reduced significantly in T2DM patients with the use of metformin in this study. These results are similar to lots of previous studies (9, 85). Research showed that heart disease, cancer, stroke, and diabetes are the major causes of death in the US population (87). Metformin, as one kind of classic first-line hypoglycemic agent, has cardiovascular protection, and it could also protect against many kinds of cancers (e.g., breast, colorectal, and prostate cancer) (88). These might lead to reduced risks of all-cause and cardiovascular mortality. However, Liu et al. (19) reported that metformin vs. diet therapy was not associated with significantly lower cardiovascular mortality in T2DM patients. Dietary therapy always refers to a low carbohydrate diet or/and a low-fat diet, which is also called a low-calorie diet (89). A low-calorie diet could reduce the risk of CVD by reducing the body weight, body mass index, fat mass, and low-density lipoprotein cholesterol levels (90, 91), which may contribute to lowing cardiovascular mortality. In T2DM patients, a low-calorie diet also could normalize insulin sensitivity and improve pancreatic beta-cell function by reducing pancreatic fat content (92, 93), which may show similar function as metformin.

The risk of hospitalization can be used to evaluate the quality of life among patients with T2DM. Studies show different opinions on whether metformin is associated with reducing the risk of hospitalization. A propensity-matched study in the community showed that metformin would remarkably reduce the hospitalization rate (50). While a 2021 retrospective cohort study found that metformin could not significantly reduce the risk of hospitalization (73). In this meta-analysis, we found metformin could not significantly reduce the risk of hospitalization in T2DM patients compared to the control group. We included 8 studies without differentiating the reasons for hospitalization, which may exist some biases. More research is needed to analyze the relationship between metformin management and the risk of hospitalization divided by different diseases.

Sulphonylurea, which has been existing for approximately 70 years, is recommended as a second-line treatment in the management of type 2 diabetes (94). Many studies have testified that compared with metformin, sulphonylurea was associated with higher risks of MACEs, heart failure, hospitalization rate, all-cause, and cardiovascular mortality (14, 71, 72). In our meta-analysis, these results were proved. Therefore, it is reasonable to recommend metformin considering its benefits above. SGLT2i and DPP-4i are relatively new hypoglycemic agents. In recent studies, these two kinds of drugs show cardiovascular benefits beyond glycemic control through anti-inflammatory pathways (95, 96). In this meta-analysis, we found there existed no significantly different effect of SGLT2i or DPP-4i vs. metformin on reducing the risk of MACEs, hospitalization, stroke, and AMI in different studies. It suggests that SGLT2i and DPP-4i may have cardiovascular protective capacity comparable to metformin. However, due to the data limitation, we couldn't make related assessments and we hope more studies could be included to find some results. In this meta-analysis, we also found metformin could reduce the risk of heart failure, and hospitalization compared with non-metformin therapy in T2DM patients with heart failure, while the difference was not significant in T2DM patients, which indicated that metformin might have higher cardiovascular protection among patients with heart failure. Related research needs to be implemented to find some mechanisms.

Our results support that metformin should be recommended as a first-line hypoglycemic drug to all T2DM patients, including those with heart failure or CKD. Because it can reduce all-cause mortality and cardiovascular mortality. Dietary management is supposed to popularize among all T2DM patients since its effect on reducing all-cause mortality has no significant difference with metformin. Metformin significantly reduced MACE, heart failure, in-hospital all-cause mortality, and cardiovascular mortality compared with sulfonylureas. Thus, we have more reasons to recommend metformin as an antidiabetic drug between these two drugs. Metformin could reduce cardiovascular mortality while it couldn't reduce the risk of MACEs, heart failure, and AMI. Perhaps metformin can reduce the severity of cardiovascular events and more studies need to testify to it. The studies on the effect of metformin monotherapy on the risk of AMI, stroke, heart failure, and hospitalization are insufficient, more multicenter studies should be implemented to guide us in the use of metformin on T2DM patients better.

### Limitations

Our meta-analysis still had several limitations. First, although we included observational studies in this study, we didn't include RCTs in this study and therefore more data from large RCTs were still needed to bring clarity to the effect of metformin on adverse outcomes in T2DM patients. Second, the comparison between metformin and SGLT2i or DPP-4i needs to be further explored because of limited data in our study in which only 2 studies were pooled for analysis, and there was still limited data focusing on comparing the long-term effect of metformin and SGLT2i or DPP-4i in T2DM patients, remaining an empty field for meta-analysis in the future. Third, significant heterogeneity with *I*^2^ > 50% was found in a major part of our data analyses with a random-effects model, of which the results should be explained cautiously.

## Conclusions

The effect of metformin on some of the adverse outcomes was not significantly better than the non-metformin therapy or DPP-4i in T2DM patients based on observational studies.

## Data availability statement

The original contributions presented in the study are included in the article/Supplementary material, further inquiries can be directed to the corresponding authors.

## Author contributions

All authors listed have made a substantial, direct, and intellectual contribution to the work and approved it for publication.

## Conflict of interest

The authors declare that the research was conducted in the absence of any commercial or financial relationships that could be construed as a potential conflict of interest. The handling editor declared a shared affiliation with the authors HZ, CW, and JJ at the time of review.

## Publisher's note

All claims expressed in this article are solely those of the authors and do not necessarily represent those of their affiliated organizations, or those of the publisher, the editors and the reviewers. Any product that may be evaluated in this article, or claim that may be made by its manufacturer, is not guaranteed or endorsed by the publisher.

## References

[1] Tsao CW Aday AW Almarzooq ZI Alonso A Beaton AZ Bittencourt MS . Heart disease and stroke statistics-2022 update: a report from the American Heart Association. Circulation. (2022) 145:e153–e639. 10.1161/CIR.0000000000001052 35078371

[2] American Diabetes Association. Improving care and promoting health in populations: standards of medical care in diabetes-2020. Diabetes Care. (2020) 43 (Suppl. 1):S7–S13. 10.2337/dc20-S001 31862744 PMC11869376

[3] Zarich SW. Antidiabetic agents and cardiovascular risk in type 2 diabetes. Nat Rev Endocrinol. (2009). 5:500–6. 10.1038/nrendo.2009.150 19636325

[4] Andersson C Vasan RS. Epidemiology of cardiovascular disease in young individuals. Nat Rev Cardiol. (2018) 15:230–40. 10.1038/nrcardio.2017.154 29022571

[5] Jiralerspong S Palla SL Giordano SH Meric-Bernstam F Liedtke C Barnett CM . Metformin and pathologic complete responses to neoadjuvant chemotherapy in diabetic patients with breast cancer. J Clin Oncol. (2009) 27:3297–302. 10.1200/JCO.2009.19.6410 19487376 PMC2736070

[6] Løvvik TS Carlsen SM Salvesen Ø Steffensen B Bixo M Gómez-Real F . Use of metformin to treat pregnant women with polycystic ovary syndrome (PregMet2): a randomised, double-blind, placebo-controlled trial. Lancet Diabetes Endocrinol. (2019) 7:256–66. 10.1016/S2213-8587(19)30002-6 30792154

[7] Ayoub R Ruddy RM Cox E Oyefiade A Derkach D Laughlin S . Assessment of cognitive and neural recovery in survivors of pediatric brain tumors in a pilot clinical trial using metformin. Nat Med. (2020) 26:1285–94. 10.1038/s41591-020-0985-2 32719487 PMC8176964

[8] Zhang K Yang W Dai H Deng Z. Cardiovascular risk following metformin treatment in patients with type 2 diabetes mellitus: results from meta-analysis. Diabetes Res Clin Pract. (2020) 160:108001. 10.1016/j.diabres.2020.108001 31904444

[9] Li T Providencia R Jiang W Liu M Yu L Gu C . Association of metformin with the mortality and incidence of cardiovascular events in patients with pre-existing cardiovascular diseases. Drugs. (2022) 82:311–22. 10.1007/s40265-021-01665-0 35032305

[10] UK Prospective Diabetes Study (UKPDS) Group. Effect of intensive blood-glucose control with metformin on complications in overweight patients with type 2 diabetes (UKPDS 34). UK Prospective Diabetes Study (UKPDS) Group. Lancet. (1998) 352:854–65. 10.1016/S0140-6736(98)07037-8 9742977

[11] Tseng CH. Metformin use is associated with a lower risk of hospitalization for heart failure in patients with type 2 diabetes mellitus: a retrospective cohort analysis. J Am Heart Assoc. (2019) 8:e011640. 10.1161/JAHA.118.011640 31630591 PMC6898844

[12] Charytan DM Solomon SD Ivanovich P Remuzzi G Cooper ME McGill JB . Metformin use and cardiovascular events in patients with type 2 diabetes and chronic kidney disease. Diabetes Obes Metab. (2019) 21:1199–208. 10.1111/dom.13642 30672083

[13] Retwiński A Kosmalski M Crespo-Leiro M Maggioni A Opolski G Ponikowski P . The influence of metformin and the presence of type 2 diabetes mellitus on mortality and hospitalisation in patients with heart failure. Kardiol Pol. (2018) 76:1336–43. 10.5603/KP.a2018.0127 29862487

[14] Filion KB Douros A Azoulay L Yin H Yu OH Suissa S. Sulfonylureas as initial treatment for type 2 diabetes and the risk of adverse cardiovascular events: a population-based cohort study. Br J Clin Pharmacol. (2019) 85:2378–89. 10.1111/bcp.14056 31276600 PMC6783602

[15] Fralick M Schneeweiss S Redelmeier DA Razak F Gomes T Patorno E. Comparative effectiveness and safety of sodium-glucose cotransporter-2 inhibitors versus metformin in patients with type 2 diabetes: an observational study using data from routine care. Diabetes Obes Metab. (2021) 23:2320–8. 10.1111/dom.14474 34169619

[16] Lee CG Heckman-Stoddard B Dabelea D Gadde KM Ehrmann D Ford L . Effect of metformin and lifestyle interventions on mortality in the diabetes prevention program and diabetes prevention program outcomes study. Diabetes Care. (2021) 44:2775–82. 10.2337/figshare.16652587.v1 34697033 PMC8669534

[17] Li T Providencia R Mu N Yin Y Chen M Wang Y . Association of metformin monotherapy or combined therapy with cardiovascular risks in patients with type 2 diabetes mellitus. Cardiovasc Diabetol. (2021) 20:30. 10.1186/s12933-020-01202-5 33516224 PMC7847575

[18] Han Y Xie H Liu Y Gao P Yang X Shen Z. Effect of metformin on all-cause and cardiovascular mortality in patients with coronary artery diseases: a systematic review and an updated meta-analysis. Cardiovasc Diabetol. (2019) 18:96. 10.1186/s12933-019-0900-7 31362743 PMC6668189

[19] Liu L Simon B Shi J Mallhi AK Eisen HJ. Impact of diabetes mellitus on risk of cardiovascular disease and all-cause mortality: evidence on health outcomes and antidiabetic treatment in United States adults. World J Diabetes. (2016) 7:449–61. 10.4239/wjd.v7.i18.449 27795819 PMC5065665

[20] Zhu W Ye Z Chen S Wu D He J Dong Y . Comparative effectiveness and safety of non-vitamin k antagonist oral anticoagulants in atrial fibrillation patients. Stroke. (2021) 52:1225–33. 10.1161/STROKEAHA.120.031007 33596677

[21] Kitao N Miyoshi H Furumoto T Ono K Nomoto H Miya A . The effects of vildagliptin compared with metformin on vascular endothelial function and metabolic parameters: a randomized, controlled trial (Sapporo Athero-Incretin Study 3). Cardiovasc Diabetol. (2017) 16:125. 10.1186/s12933-017-0607-6 29017497 PMC5634845

[22] Wong AK Symon R AlZadjali MA Ang DS Ogston S Choy A . The effect of metformin on insulin resistance and exercise parameters in patients with heart failure. Eur J Heart Fail. (2012) 14:1303–10. 10.1093/eurjhf/hfs106 22740509

[23] Al Ali L Hartman MT Lexis CP Hummel YM Lipsic E van Melle JP . The effect of metformin on diastolic function in patients presenting with ST-elevation myocardial infarction. PLoS ONE. (2016) 11:e0168340. 10.1371/journal.pone.0168340 27977774 PMC5158040

[24] Lexis CP van der Horst-Schrivers AN Lipsic E Valente MA Muller Kobold AC de Boer RA . The effect of metformin on cardiovascular risk profile in patients without diabetes presenting with acute myocardial infarction: data from the glycometabolic intervention as adjunct to primary coronary intervention in ST elevation myocardial infarction (GIPS-III) trial. BMJ Open Diabetes Res Care. (2015) 3:e000090. 10.1136/bmjdrc-2015-000090 26688733 PMC4679814

[25] Eroglu TE Jia L Blom MT Verkerk AO Devalla HD Boink GJJ . Sulfonylurea antidiabetics are associated with lower risk of out-of-hospital cardiac arrest: real-world data from a population-based study. Br J Clin Pharmacol. (2021) 87:3588–98. 10.1111/bcp.14774 33896015 PMC8453495

[26] Komaru Y Takeuchi T Suzuki L Asano T Urayama KY. Recurrent cardiovascular events in patients with newly diagnosed acute coronary syndrome: influence of diabetes and its management with medication. J Diabetes Complications. (2020) 34:107511. 10.1016/j.jdiacomp.2019.107511 31928892

[27] Lexis CP van der Horst IC Lipsic E van der Harst P van der Horst-Schrivers AN Wolffenbuttel BH . Metformin in non-diabetic patients presenting with ST elevation myocardial infarction: rationale and design of the glycometabolic intervention as adjunct to primary percutaneous intervention in ST elevation myocardial infarction (GIPS)-III trial. Cardiovasc Drugs Ther. (2012) 26:417–26. 10.1007/s10557-012-6413-1 22968678 PMC3464381

[28] Preiss D Lloyd SM Ford I McMurray JJ Holman RR Welsh P . Metformin for non-diabetic patients with coronary heart disease (the CAMERA study): a randomised controlled trial. Lancet Diabetes Endocrinol. (2014) 2:116–24. 10.1016/S2213-8587(13)70152-9 24622715

[29] Basnet S Kozikowski A Makaryus AN Pekmezaris R Zeltser R Akerman M . Metformin and myocardial injury in patients with diabetes and st-segment elevation myocardial infarction: a propensity score matched analysis. J Am Heart Assoc. (2015) 4:e002314. 10.1161/JAHA.115.002314 26494519 PMC4845135

[30] Zeller M Labalette-Bart M Juliard JM Potier L Feldman LJ Steg PG . Metformin and contrast-induced acute kidney injury in diabetic patients treated with primary percutaneous coronary intervention for ST segment elevation myocardial infarction: amulticenter study. Int J Cardiol. (2016) 220:137–42. 10.1016/j.ijcard.2016.06.076 27376570

[31] Kooy A de Jager J Lehert P Bets D Wulffelé MG Donker AJ . Long-term effects of metformin on metabolism and microvascular and macrovascular disease in patients with type 2 diabetes mellitus. Arch Intern Med. (2009) 169:616–25. 10.1001/archinternmed.2009.20 19307526

[32] Liu Y Jiang X Chen X. Liraglutide and Metformin alone or combined therapy for type 2 diabetes patients complicated with coronary artery disease. Lipids Health Dis. (2017) 16:227. 10.1186/s12944-017-0609-0 29197387 PMC5712174

[33] Fauchier G Bisson A Bodin A Herbert J Angoulvant D Ducluzeau PH . Glucose-lowering drug use and new-onset atrial fibrillation in patients with diabetes mellitus. Diabetologia. (2021) 64:2602–5. 10.1007/s00125-021-05551-y 34435218

[34] Halabi A Yang H Wright L Potter E Huynh Q Negishi K . Evolution of myocardial dysfunction in asymptomatic patients at risk of heart failure. JACC Cardiovasc Imaging. (2021) 14:350–61. 10.1016/j.jcmg.2020.09.032 33221236

[35] Ono K Wada H Satoh-Asahara N Inoue H Uehara K Funada J . Effects of metformin on left ventricular size and function in hypertensive patients with type 2 diabetes mellitus: results of a randomized, controlled, multicenter, phase IV trial. Am J Cardiovasc Drugs. (2020) 20:283–293. 10.1007/s40256-019-00381-1 31721026 PMC7266803

[36] Deshmukh A Ghannam M Liang J Saeed M Cunnane R Ghanbari H . Effect of metformin on outcomes of catheter ablation for atrial fibrillation. J Cardiovasc Electrophysiol. (2021) 32:1232–9. 10.1111/jce.14954 33600005

[37] Kruszelnicka O Chyrchel B Golay A Surdacki A. Differential associations of circulating asymmetric dimethylarginine and cell adhesion molecules with metformin use in patients with type 2 diabetes mellitus and stable coronary artery disease. Amino Acids. (2015) 47:1951–9. 10.1007/s00726-015-1976-3 25859650

[38] Lexis CP Wieringa WG Hiemstra B van Deursen VM Lipsic E van der Harst P . Chronic metformin treatment is associated with reduced myocardial infarct size in diabetic patients with ST-segment elevation myocardial infarction. Cardiovasc Drugs Ther. (2014) 28:163–71. 10.1007/s10557-013-6504-7 24292206

[39] Lu Y Wang Y Weng T Chen Z Sun X Wei J . Association between metformin use and coronary artery calcification in type 2 diabetic patients. J Diabetes Res. (2019) 2019:9484717. 10.1155/2019/9484717 31192264 PMC6525896

[40] Mohan M Al-Talabany S McKinnie A Mordi IR Singh JSS Gandy SJ . A randomized controlled trial of metformin on left ventricular hypertrophy in patients with coronary artery disease without diabetes: the MET-REMODEL trial. Eur Heart J. (2019) 40:3409–3417. 10.1093/eurheartj/ehz203 30993313 PMC6823615

[41] Lexis CP van der Horst IC Lipsic E Wieringa WG de Boer RA van den Heuvel AF . Effect of metformin on left ventricular function after acute myocardial infarction in patients without diabetes: the GIPS-III randomized clinical trial. Jama. (2014) 311:1526–35. 10.1001/jama.2014.3315 24687169

[42] Hartman MHT Prins JKB Schurer RAJ Lipsic E Lexis CPH van der Horst-Schrivers ANA . Two-year follow-up of 4 months metformin treatment vs. placebo in ST-elevation myocardial infarction: data from the GIPS-III RCT. Clin Res Cardiol. (2017) 106:939–46. 10.1007/s00392-017-1140-z 28755285 PMC5696505

[43] Sundström J Kristófi R Östlund O Bennet L Eliasson B Jansson S . A registry-based randomised trial comparing an SGLT2 inhibitor and metformin as standard treatment of early stage type 2 diabetes (SMARTEST): rationale, design and protocol. J Diabetes Complications. (2021) 35:107996. 10.1016/j.jdiacomp.2021.107996 34389234

[44] Dong YH Wang SV Gagne JJ Wu LC Chang CH. Comparison of different case-crossover variants in handling exposure-time trend or persistent-user bias: using dipeptidyl peptidase-4 inhibitors and the risk of heart failure as an example. Value Health. (2020) 23:217–26. 10.1016/j.jval.2019.09.2746 32113627

[45] Crowley MJ McGuire DK Alexopoulos AS Jensen TJ Rasmussen S Saevereid HA . Effects of liraglutide on cardiovascular outcomes in type 2 diabetes patients with and without baseline metformin use: post hoc analyses of the LEADER trial. Diabetes Care. (2020) 43:e108–110. 10.2337/dc20-0437 32647053 PMC7440910

[46] Sillars B Davis WA Hirsch IB Davis TM. Sulphonylurea-metformin combination therapy, cardiovascular disease and all-cause mortality: the Fremantle Diabetes Study. Diabetes Obes Metab. (2010) 12:757–65. 10.1111/j.1463-1326.2010.01230.x 20649627

[47] Evans JM Ogston SA Emslie-Smith A Morris AD. Risk of mortality and adverse cardiovascular outcomes in type 2 diabetes: a comparison of patients treated with sulfonylureas and metformin. Diabetologia. (2006) 49:930–6. 10.1007/s00125-006-0176-9 16525843

[48] Jong CB Chen KY Hsieh MY Su FY Wu CC Voon WC . Metformin was associated with lower all-cause mortality in type 2 diabetes with acute coronary syndrome: a Nationwide registry with propensity score-matched analysis. Int J Cardiol. (2019) 291:152–57. 10.1016/j.ijcard.2019.03.021 30905518

[49] Bromage DI Godec TR Pujades-Rodriguez M Gonzalez-Izquierdo A Denaxas S Hemingway H . Metformin use and cardiovascular outcomes after acute myocardial infarction in patients with type 2 diabetes: a cohort study. Cardiovasc Diabetol. (2019) 18:168. 10.1186/s12933-019-0972-4 31815634 PMC6900858

[50] Romero SP Andrey JL Garcia-Egido A Escobar MA Perez V Corzo R . Metformin therapy and prognosis of patients with heart failure and new-onset diabetes mellitus. A propensity-matched study in the community. Int J Cardiol. (2013) 166:404–12. 10.1016/j.ijcard.2011.10.141 22112681

[51] Fácila L Fabregat-Andrés Ó Bertomeu V Navarro JP Miñana G García-Blas S . Metformin and risk of long-term mortality following an admission for acute heart failure. J Cardiovasc Med (Hagerstown). (2017) 18:69–73. 10.2459/JCM.0000000000000420 27341193

[52] Ritsinger V Lagerqvist B Lundman P Hagström E Norhammar A. Diabetes, metformin and glucose lowering therapies after myocardial infarction: insights from the SWEDEHEART registry. Diab Vasc Dis Res. (2020) 17:1479164120973676. 10.1177/1479164120973676 33231125 PMC7919225

[53] Pantalone KM Kattan MW Yu C Wells BJ Arrigain S Jain A . The risk of developing coronary artery disease or congestive heart failure, overall mortality. In type 2 diabetic patients receiving rosiglitazone, pioglitazone, metformin, or sulfonylureas: a retrospective analysis. Acta Diabetol. (2009) 46:145–54. 10.1007/s00592-008-0090-3 19194648

[54] Jung I Kwon H Park SE Han KD Park YG Rhee EJ . The effects of glucose lowering agents on the secondary prevention of coronary artery disease in patients with type 2 diabetes. Endocrinol Metab (Seoul). (2021) 36:977–87. 10.3803/EnM.2021.1046 34645126 PMC8566121

[55] Abualsuod A Rutland JJ Watts TE Pandat S Delongchamp R Mehta JL. The effect of metformin use on left ventricular ejection fraction and mortality post-myocardial infarction. Cardiovasc Drugs Ther. (2015) 29:265–75. 10.1007/s10557-015-6601-x 26068409

[56] Wang J Lu Y Min X Yuan T Wei J Cai Z. The association between metformin treatment and outcomes in type 2 diabetes mellitus patients with heart failure with preserved ejection fraction: a retrospective study. Front Cardiovasc Med. (2021) 8:648212. 10.3389/fcvm.2021.648212 33778026 PMC7994337

[57] Chen TH Li YR Chen SW Lin YS Sun CC Chen DY . Sodium-glucose cotransporter 2 inhibitor versus metformin as first-line therapy in patients with type 2 diabetes mellitus: a multi-institution database study. Cardiovasc Diabetol. (2020) 19:189. 10.1186/s12933-020-01169-3 33167990 PMC7654060

[58] Chen CB Lin M Eurich DT Johnson JA. Safety of concomitant metformin and proton pump inhibitor use: a population retrospective cohort study. Clin Ther. (2016) 38:1392–400. 10.1016/j.clinthera.2016.03.024 27061884

[59] He S Qian X Chen Y Shen X Zhang B Chen X . Risk of death and heart failure among patients with type 2 diabetes treated by metformin and nonmetformin monotherapy: a real-world study. J Diabetes Res. (2021) 2021:5534387. 10.1155/2021/5534387 34222493 PMC8213465

[60] Johnson JA Simpson SH Toth EL Majumdar SR. Reduced cardiovascular morbidity and mortality associated with metformin use in subjects with Type 2 diabetes. Diabet Med. (2005) 22:497–502. 10.1111/j.1464-5491.2005.01448.x 15787679

[61] Duncan AI Koch CG Xu M Manlapaz M Batdorf B Pitas G . Recent metformin ingestion does not increase in-hospital morbidity or mortality after cardiac surgery. Anesth Analg. (2007) 104:42–50. 10.1213/01.ane.0000242532.42656.e7 17179241

[62] Schramm TK Gislason GH Vaag A Rasmussen JN Folke F Hansen ML . Mortality and cardiovascular risk associated with different insulin secretagogues compared with metformin in type 2 diabetes, with or without a previous myocardial infarction: a nationwide study. Eur Heart J. (2011) 32:1900–8. 10.1093/eurheartj/ehr077 21471135

[63] Tseng CH. Metformin use is associated with a lower incidence of hospitalization for atrial fibrillation in patients with type 2 diabetes mellitus. Front Med (Lausanne). (2020) 7:592901. 10.3389/fmed.2020.592901 33693008 PMC7937645

[64] Roussel R Travert F Pasquet B Wilson PW Smith Jr SC Goto S . Metformin use and mortality among patients with diabetes and atherothrombosis. Arch Intern Med. (2010) 170:1892–9. 10.1001/archinternmed.2010.409 21098347

[65] Kim MH Oh HJ Kwon SH Jeon JS Noh H Han DC . Metformin use and cardiovascular outcomes in patients with diabetes and chronic kidney disease: a nationwide cohort study. Kidney Res Clin Pract. (2021) 40:660–72. 10.23876/j.krcp.20.222 34922433 PMC8685353

[66] Shah DD Fonarow GC Horwich TB. Metformin therapy and outcomes in patients with advanced systolic heart failure and diabetes. J Card Fail. (2010) 16:200–6. 10.1016/j.cardfail.2009.10.022 20206893 PMC2855621

[67] Richardson TL Jr., Hackstadt AJ, Hung AM, Greevy RA, Grijalva CG, et al. Hospitalization for heart failure among patients with diabetes mellitus and reduced kidney function treated with metformin versus sulfonylureas: a retrospective cohort study. J Am Heart Assoc. (2021) 10:e019211. 10.1161/JAHA.120.019211 33821674 PMC8174186

[68] Fung CS Wan EY Wong CK Jiao F Chan AK. Effect of metformin monotherapy on cardiovascular diseases and mortality: a retrospective cohort study on Chinese type 2 diabetes mellitus patients. Cardiovasc Diabetol. (2015) 14:137. 10.1186/s12933-015-0304-2 26453464 PMC4600251

[69] Baksh SN Segal JB McAdams-DeMarco M Kalyani RR Alexander GC Ehrhardt S. Dipeptidyl peptidase-4 inhibitors and cardiovascular events in patients with type 2 diabetes, without cardiovascular or renal disease. PLoS One. (2020) 15:e0240141. 10.1371/journal.pone.0240141 33057387 PMC7561135

[70] Wang CP Lorenzo C Habib SL Jo B Espinoza SE. Differential effects of metformin on age related comorbidities in older men with type 2 diabetes. J Diabetes Complications. (2017) 31:679–86. 10.1016/j.jdiacomp.2017.01.013 28190681 PMC5654524

[71] Roumie CL Min JY D'Agostino McGowan L Presley C Grijalva CG Hackstadt AJ . Comparative safety of sulfonylurea and metformin monotherapy on the risk of heart failure: a cohort study. J Am Heart Assoc. (2017) 6:5379. 10.1161/JAHA.116.005379 28424149 PMC5533028

[72] Roumie CL Hung AM Greevy RA Grijalva CG Liu X Murff HJ . Comparative effectiveness of sulfonylurea and metformin monotherapy on cardiovascular events in type 2 diabetes mellitus: a cohort study. Ann Intern Med. (2012) 157:601–10. 10.7326/0003-4819-157-9-201211060-00003 23128859 PMC4667563

[73] Khan MS Solomon N DeVore AD Sharma A Felker GM Hernandez AF . Clinical outcomes with metformin and sulfonylurea therapies among patients with heart failure and diabetes. JACC Heart Fail. (2022) 10:198–210. 10.1016/j.jchf.2021.11.001 34895861

[74] Clegg LE Jing Y Penland RC Boulton DW Hernandez AF Holman RR . Cardiovascular and renal safety of metformin in patients with diabetes and moderate or severe chronic kidney disease: Observations from the EXSCEL and SAVOR-TIMI 53 cardiovascular outcomes trials. Diabetes Obes Metab. (2021) 23:1101–10. 10.1111/dom.14313 33394543

[75] Roumie CL Chipman J Min JY Hackstadt AJ Hung AM Greevy Jr RA . Association of treatment with metformin vs sulfonylurea with major adverse cardiovascular events among patients with diabetes and reduced kidney function. Jama. (2019) 322:1167–77. 10.1001/jama.2019.13206 31536102 PMC6753652

[76] Gu J Yin ZF Zhang JF Wang CQ. Association between long-term prescription of metformin and the progression of heart failure with preserved ejection fraction in patients with type 2 diabetes mellitus and hypertension. Int J Cardiol. (2020) 306:140–5. 10.1016/j.ijcard.2019.11.087 31711850

[77] Morgan CL Mukherjee J Jenkins-Jones S Holden SE Currie CJ. Association between first-line monotherapy with sulphonylurea versus metformin and risk of all-cause mortality and cardiovascular events: a retrospective, observational study. Diabetes Obes Metab. (2014) 16:957–62. 10.1111/dom.12302 24720708

[78] Scheller NM Mogensen UM Andersson C Vaag A Torp-Pedersen C. All-cause mortality and cardiovascular effects associated with the DPP-IV inhibitor sitagliptin compared with metformin, a retrospective cohort study on the Danish population. Diabetes Obes Metab. (2014) 16:231–6. 10.1111/dom.12197 24020750

[79] Whitlock RH Hougen I Komenda P Rigatto C Clemens KK Tangri N. A safety comparison of metformin vs sulfonylurea initiation in patients with type 2 diabetes and chronic kidney disease: a retrospective cohort study. Mayo Clin Proc. (2020) 95:90–100. 10.1016/j.mayocp.2019.07.017 31902433

[80] Intensive blood-glucose control with sulphonylureas or insulin compared with conventional treatment and risk of complications in patients with type 2 diabetes (UKPDS 33). UK Prospective Diabetes Study (UKPDS) Group. Lancet. (1998) 352:837–53. 10.1016/S0140-6736(98)07019-6 9742976

[81] Sposito AC Breder I Soares AAS Kimura-Medorima ST Munhoz DB Cintra RMR . Dapagliflozin effect on endothelial dysfunction in diabetic patients with atherosclerotic disease: a randomized active-controlled trial. Cardiovasc Diabetol. (2021) 20:74. 10.1186/s12933-021-01264-z 33771149 PMC8004411

[82] Shen Y Ding FH Dai Y Wang XQ Zhang RY Lu L . Reduced coronary collateralization in type 2 diabetic patients with chronic total occlusion. Cardiovasc Diabetol. (2018) 17:26. 10.1186/s12933-018-0671-6 29422093 PMC5804044

[83] Gao J Yuan J Wang Q Lei T Shen X Cui B . Metformin protects against PM (2.5)-induced lung injury and cardiac dysfunction independent of AMP-activated protein kinase α2. Redox Biol. (2020) 28:101345. 10.1016/j.redox.2019.101345 31669973 PMC6838896

[84] Wang Q Zhang M Torres G Wu S Ouyang C Xie Z . Metformin suppresses diabetes-accelerated atherosclerosis via the inhibition of drp1-mediated mitochondrial fission. Diabetes. (2017) 66:193–205. 10.2337/db16-0915 27737949 PMC5204316

[85] Monami M Candido R Pintaudi B Targher G Mannucci E. Effect of metformin on all-cause mortality and major adverse cardiovascular events: an updated meta-analysis of randomized controlled trials. Nutr Metab Cardiovasc Dis. (2021) 31:699–704. 10.1016/j.numecd.2020.11.031 33549430

[86] Griffin SJ Leaver JK Irving GJ. Impact of metformin on cardiovascular disease: a meta-analysis of randomised trials among people with type 2 diabetes. Diabetologia. (2017) 60:1620–9. 10.1007/s00125-017-4337-9 28770324 PMC5552849

[87] Ma J Ward EM Siegel RL Jemal A. Temporal trends in mortality in the United States, 1969-2013. Jama. (2015) 314:1731–9. 10.1001/jama.2015.12319 26505597

[88] Coyle C Cafferty FH Vale C Langley RE. Metformin as an adjuvant treatment for cancer: a systematic review and meta-analysis. Ann Oncol. (2016) 27:2184–95. 10.1093/annonc/mdw410 27681864 PMC5178140

[89] Forouhi NG Misra A Mohan V Taylor R Yancy W. Dietary and nutritional approaches for prevention and management of type 2 diabetes. Bmj. (2018) 361:k2234. 10.1136/bmj.k2234 29898883 PMC5998736

[90] Sofi F Dinu M Pagliai G Cesari F Gori AM Sereni A . Low-calorie vegetarian versus mediterranean diets for reducing body weight and improving cardiovascular risk profile: CARDIVEG study (cardiovascular prevention with vegetarian diet). Circulation. (2018) 137:1103–13. 10.1161/CIRCULATIONAHA.117.030088 29483085

[91] Srour B Fezeu LK Kesse-Guyot E Allès B Méjean C Andrianasolo RM . Ultra-processed food intake and risk of cardiovascular disease: prospective cohort study (NutriNet-Santé). Bmj. (2019) 365:l1451. 10.1136/bmj.l1451 31142457 PMC6538975

[92] Lim EL Hollingsworth KG Aribisala BS Chen MJ Mathers JC Taylor R. Reversal of type 2 diabetes: normalisation of beta cell function in association with decreased pancreas and liver triacylglycerol. Diabetologia. (2011) 54:2506–14. 10.1007/s00125-011-2204-7 21656330 PMC3168743

[93] White MG Shaw JA Taylor R. Type 2 diabetes: the pathologic basis of reversible β-Cell dysfunction. Diabetes Care. (2016) 39:2080–8. 10.2337/dc16-0619 27926891

[94] Groop LC Sulfonylureas in NIDDM. Diabetes Care. (1992) 15:737–54. 10.2337/diacare.15.6.737 1600834

[95] Tomovic K Lazarevic J Kocic G Deljanin-Ilic M Anderluh M Smelcerovic A. Mechanisms and pathways of anti-inflammatory activity of DPP-4 inhibitors in cardiovascular and renal protection. Med Res Rev. (2019) 39:404–22. 10.1002/med.21513 29806214

[96] Cowie MR Fisher M. SGLT2 inhibitors: mechanisms of cardiovascular benefit beyond glycaemic control. Nat Rev Cardiol. (2020) 17:761–72. 10.1038/s41569-020-0406-8 32665641

