# Supplementary material for: Effect of metformin on adverse outcomes in T2DM patients: Systemic review and meta-analysis of observational studies
Source: Front Cardiovasc Med. 2022 Sep 23;9:944902. doi: 10.3389/fcvm.2022.944902 (PMC9539433; doi:10.3389/fcvm.2022.944902)
Supplement: Supplementary file 1 [file Data_Sheet_1.docx]

| **Outcomes** | **Patients characteristics** | **Comparison** | **No. of studies included** | **Statistic indicators** | **RR/HR and 95% CIs** | **P value** | **I^2^**  **statistics** |
| --- | --- | --- | --- | --- | --- | --- | --- |
| MACE | T2DM | metformin vs non-metformin | 4 | HR | 1.06 [0.91, 1.22] | 0.46 | 82% |
| MACE | T2DM | metformin vs sulphonylurea | 5 | HR | 0.83 [0.77, 0.90] | <0.00001 | 48% |
| MACE | T2DM | metformin vs DPP-4i | 2 | HR | 0.95[0.73, 1.23] | 0.71 | 84% |
| All-cause mortality | T2DM | metformin vs non-metformin | 12 | HR | 0.82 [0.77, 0.88] | <0.00001 | 73% |
| All-cause mortality | T2DM | metformin vs sulphonylurea | 4 | HR | 0.58 [0.49, 0.68] | <0.00001 | 74% |
| All-cause mortality | T2DM | metformin vs diet | 3 | HR | 0.76 [0.64, 0.90] | 0.002 | 0% |
| All-cause mortality | T2DM+HF | metformin vs non-metformin | 7 | HR | 0.84 [0.80, 0.88] | <0.00001 | 40% |
| All-cause mortality | T2DM+CKD | metformin vs non-metformin | 3 | HR | 0.79 [0.75, 0.82] | <0.00001 | 0% |
| Hospitalization | T2DM | metformin vs non-metformin | 8 | HR | 0.85 [0.64, 1.13] | 0.26 | 98% |
| Hospitalization | T2DM | metformin vs SGLT-2i | 2 | HR | 1.42[0.87, 2.32] | 0.16 | 49% |
| Hospitalization | T2DM+HF | metformin vs non-metformin | 4 | HR | 0.86 [0.78, 0.95] | 0.002 | 53% |
| Hospitalization | T2DM | metformin vs sulphonylurea | 4 | HR | 0.83 [0.78, 0.88] | <0.00001 | 40% |
| HF | T2DM | metformin vs non-metformin | 6 | HR | 0.86 [0.60, 1.25] | 0.44 | 99% |
| HF | T2DM | metformin vs sulphonylurea | 4 | HR | 0.80 [0.76, 0.85] | <0.00001 | 0% |
| Repeat HF | T2DM+HF | metformin vs non-metformin | 3 | HR | 0.82 [0.76, 0.87] | <0.00001 | 7% |
| Cardiovascular mortality | T2DM | metformin vs non-metformin | 6 | HR | 0.83 [0.70, 0.98] | 0.03 | 85% |
| Cardiovascular mortality | T2DM+HF | metformin vs non-metformin | 3 | HR | 0.78 [0.74, 0.82] | <0.00001 | 0% |
| Cardiovascular mortality | T2DM | metformin vs sulphonylurea | 4 | HR | 0.70 [0.58, 0.84] | 0.0001 | 0% |
| Stroke | T2DM | metformin vs non-metformin | 3 | HR | 1.16 [0.88, 1.53] | 0.30 | 84% |
| Stroke | T2DM | metformin vs SGLT-2i | 2 | HR | 1.03[0.65, 1.63] | 0.89 | 87% |
| AMI | T2DM | metformin vs non-metformin | 3 | HR | 0.88 [0.69, 1.14] | 0.34 | 88% |

MACE=Major Adverse Cardiovascular Events; HF=Heart Failure; AMI=Acute Myocardial Infarction; T2DM=Type 2 Diabetes Mellitus; CKD=Chronic Kidney Disease; DPP-4i= Dipeptidyl Peptidase-4 inhibitor; SGLT-2i=Sodium-dependent Glucose Transporter-2 inhibitor; HR=Hazard Ratio; CI=Confidence Interval

**Supplementary Table 1. Summary of the outcomes**

| **Outcomes** | **Patients characteristics** | **Comparison** | **No. of studies included** | **Statistic indicators** | **RR/HR and 95% CIs** | **P value** | **I^2^**  **statistics** |
| --- | --- | --- | --- | --- | --- | --- | --- |
| MACE | T2DM | metformin vs non-metformin | 3 | HR | 1.09 [0.96, 1.23] | 0.18 | 82% |
| MACE | T2DM | metformin vs sulphonylurea | 4 | HR | 0.83 [0.76, 0.92] | 0.0003 | 61% |
| MACE | T2DM | metformin vs DPP-4i | 2 | HR | 0.95[0.73, 1.23] | 0.71 | 84% |
| All-cause mortality | T2DM | metformin vs non-metformin | 5 | HR | 0.79 [0.68, 0.91] | 0.001 | 84% |
| All-cause mortality | T2DM | metformin vs sulphonylurea | 4 | HR | 0.58 [0.49, 0.68] | <0.00001 | 74% |
| All-cause mortality | T2DM | metformin vs diet | 3 | HR | 0.76 [0.64, 0.90] | 0.002 | 0% |
| All-cause mortality | T2DM+HF | metformin vs non-metformin | 1 | - | - | - | - |
| All-cause mortality | T2DM+CKD | metformin vs non-metformin | 3 | HR | 0.79 [0.75, 0.82] | <0.00001 | 0% |
| Hospitalization | T2DM | metformin vs non-metformin | 4 | HR | 0.73 [0.48, 1.12] | 0.15 | 96% |
| Hospitalization | T2DM | metformin vs SGLT-2i | 2 | HR | 1.42[0.87, 2.32] | 0.16 | 49% |
| Hospitalization | T2DM+HF | metformin vs non-metformin | 0 | - | - | - | - |
| Hospitalization | T2DM+HF | metformin vs sulphonylurea | 4 | HR | 0.83 [0.77, 0.89] | <0.00001 | 57% |
| HF | T2DM | metformin vs non-metformin | 3 | HR | 0.73 [0.48, 1.13] | 0.16 | 97% |
| HF | T2DM | metformin vs sulphonylurea | 3 | HR | 0.80 [0.74, 0.86] | <0.00001 | 30% |
| Repeat HF | T2DM+HF | metformin vs non-metformin | 0 | - | - | - | - |
| Cardiovascular mortality | T2DM | metformin vs non-metformin | 4 | HR | 0.85 [0.69, 1.06] | 0.15 | 78% |
| Cardiovascular mortality | T2DM+HF | metformin vs non-metformin | 1 | - | - | - | - |
| Cardiovascular mortality | T2DM | metformin vs sulphonylurea | 2 | HR | 0.66 [0.49, 0.89] | 0.007 | 0% |
| Stroke | T2DM | metformin vs non-metformin | 3 | HR | 1.16 [0.88, 1.53] | 0.3 | 84% |
| Stroke | T2DM | metformin vs SGLT-2i | 2 | HR | 1.03[0.65, 1.63] | 0.89 | 87% |
| AMI | T2DM | metformin vs non-metformin | 3 | HR | 0.88 [0.69, 1.14] | 0.34 | 88% |

MACE=Major Adverse Cardiovascular Events; HF=Heart Failure; AMI=Acute Myocardial Infarction; T2DM=Type 2 Diabetes Mellitus; CKD=Chronic Kidney Disease; DPP-4i= Dipeptidyl Peptidase-4 inhibitor; SGLT-2i=Sodium-dependent Glucose Transporter-2 inhibitor; HR=Hazard Ratio; CI=Confidence Interval

**Supplementary Table 2. Sensitivity analysis for the outcomes**


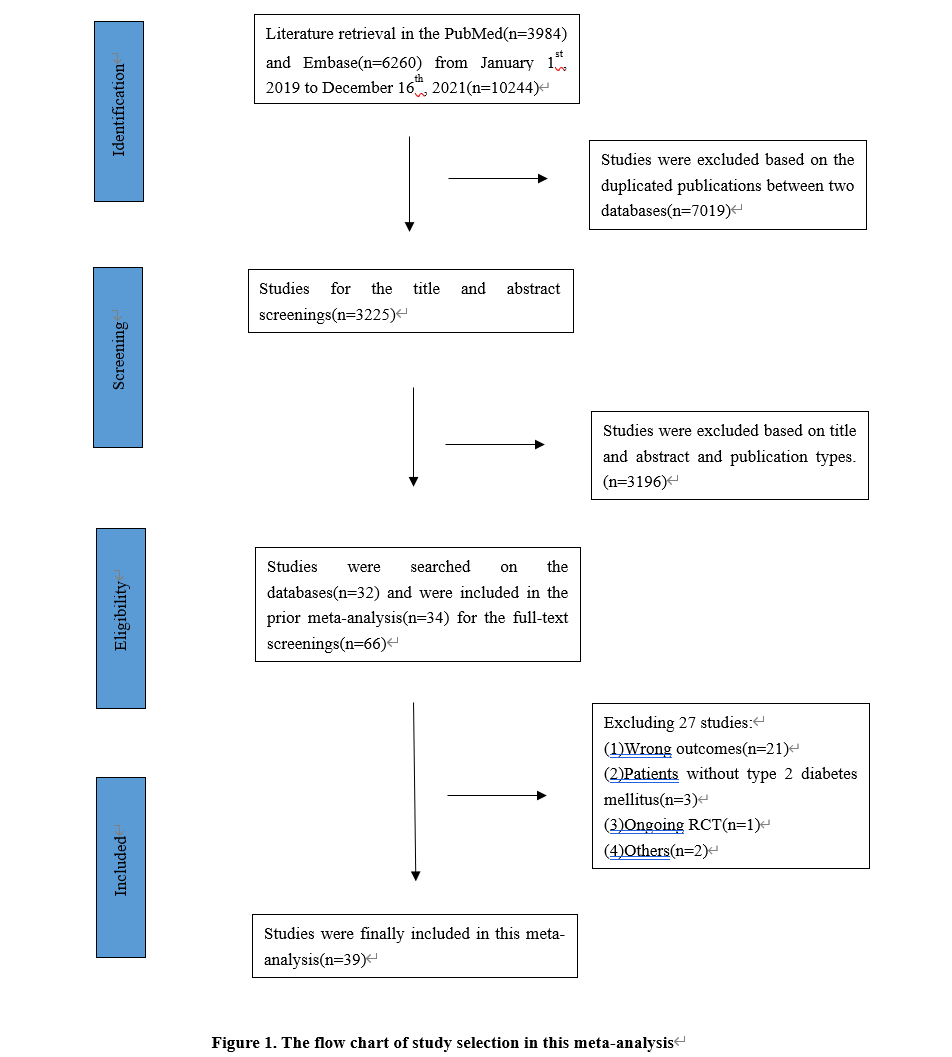


**Supplementary Figure 1. The flow chart of study selection in this meta-analysis**


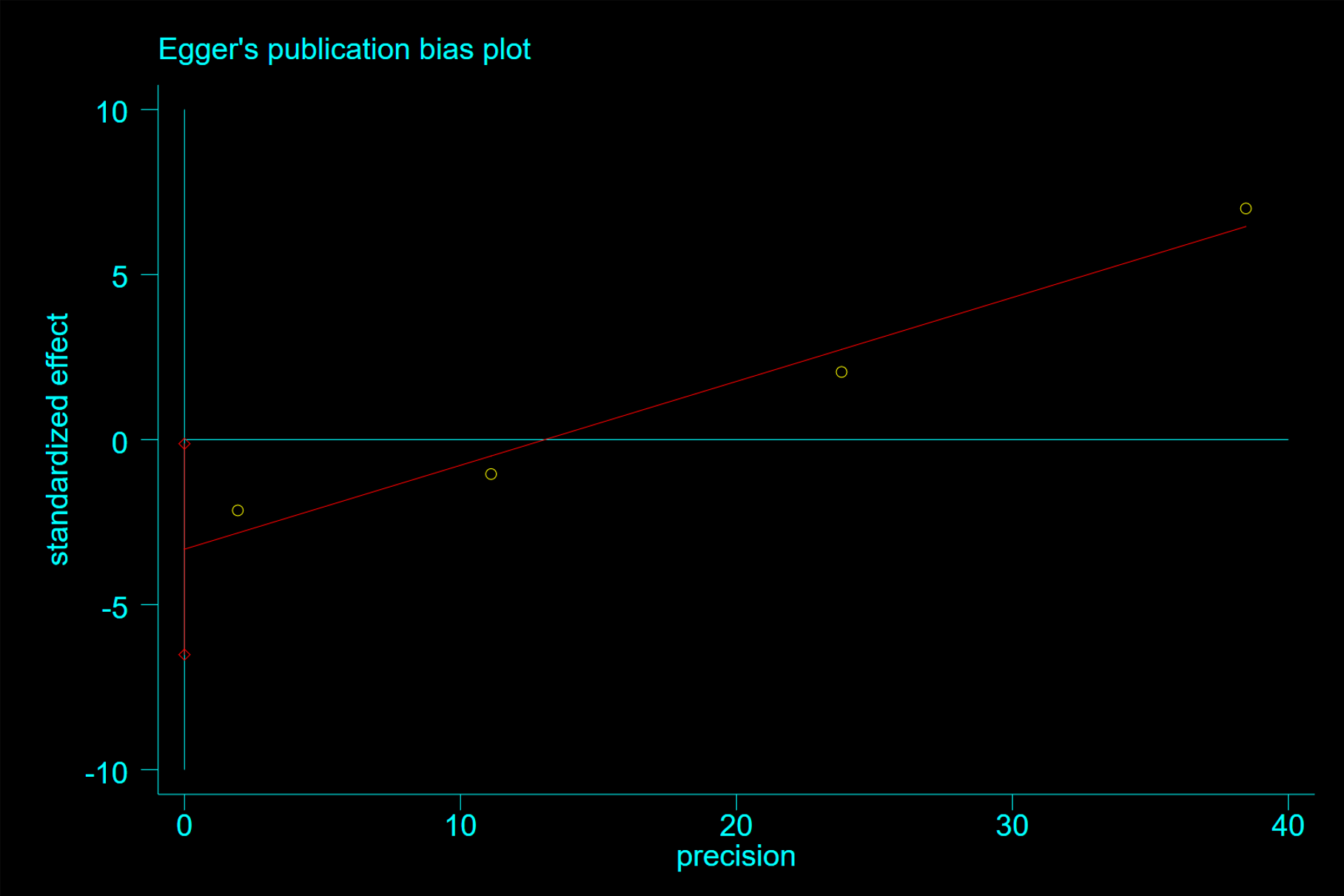


**Supplementary Figure 2**. Egger test(P=0.047) for hazard ratio of MACE among patients with metformin therapy vs non-metformin therapy

Note: Begg’s test(P=0.308)


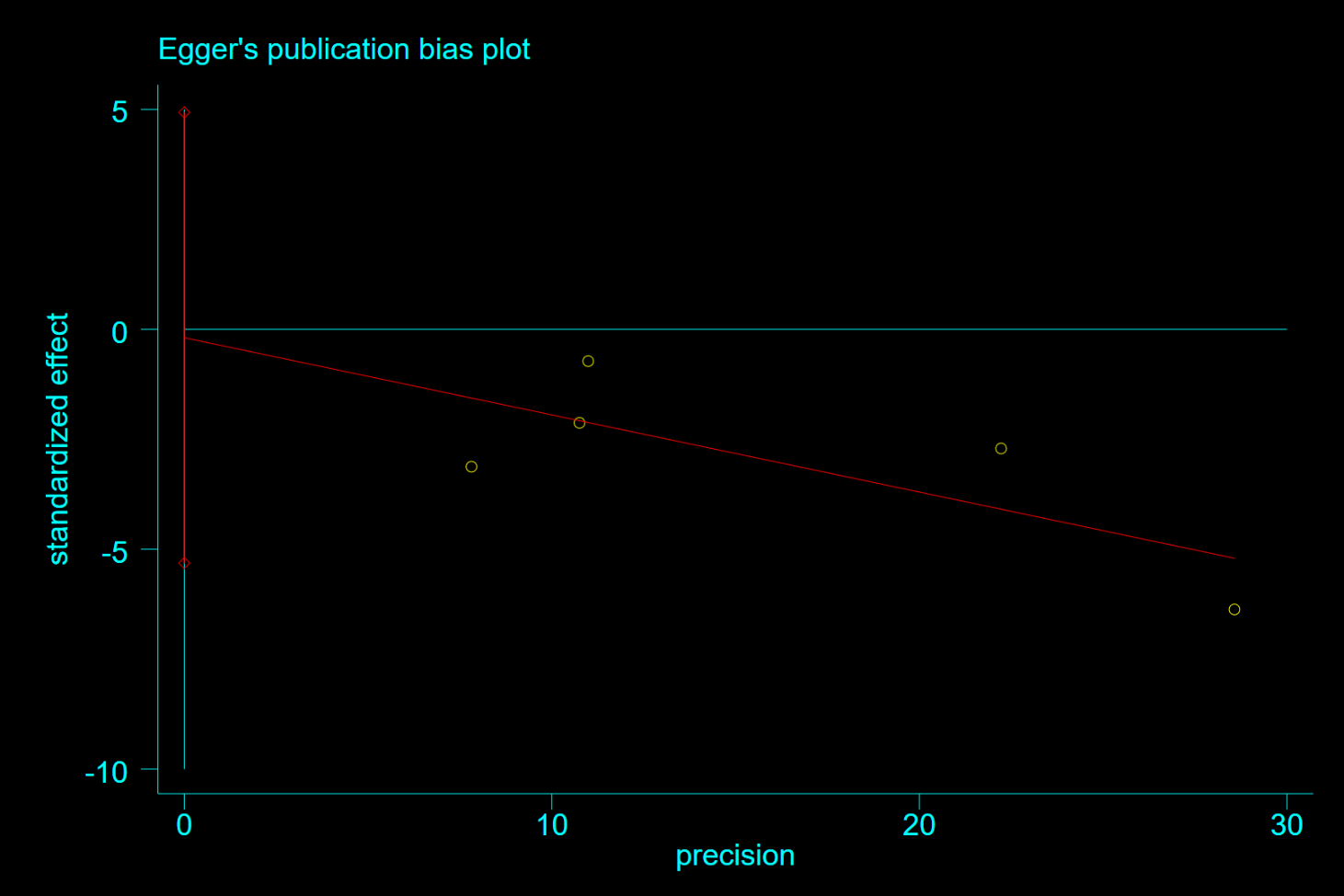


**Supplementary Figure 3**. Egger test(P=0.912) for hazard ratio of MACE among patients with metformin therapy vs sulphonylurea therapy

Note: Begg test(P=0.462)


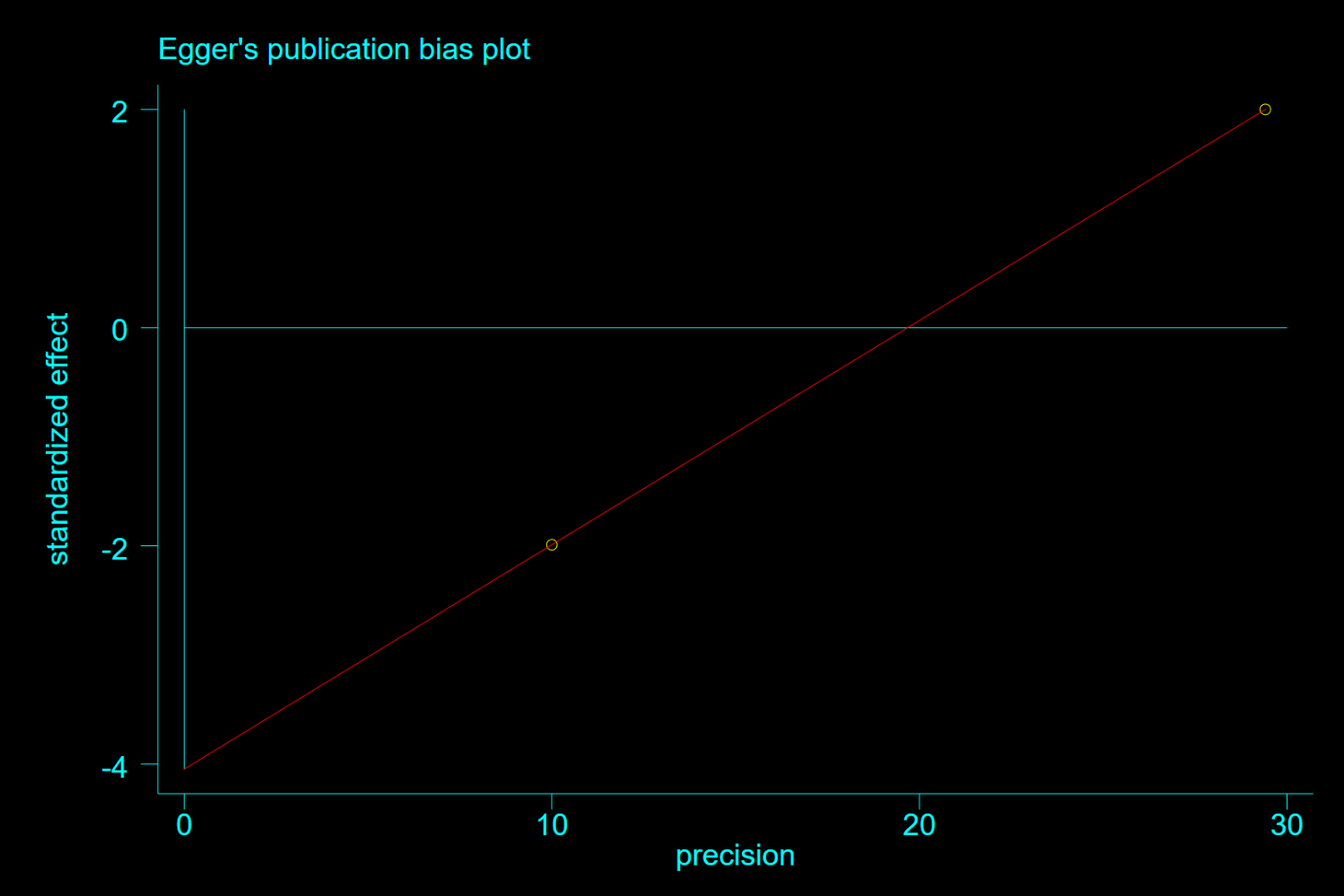


**Supplementary Figure 4**. Egger test for hazard ratio of MACE among patients with metformin therapy vs DPP-4i therapy

Note: Begg test(P=1.0)


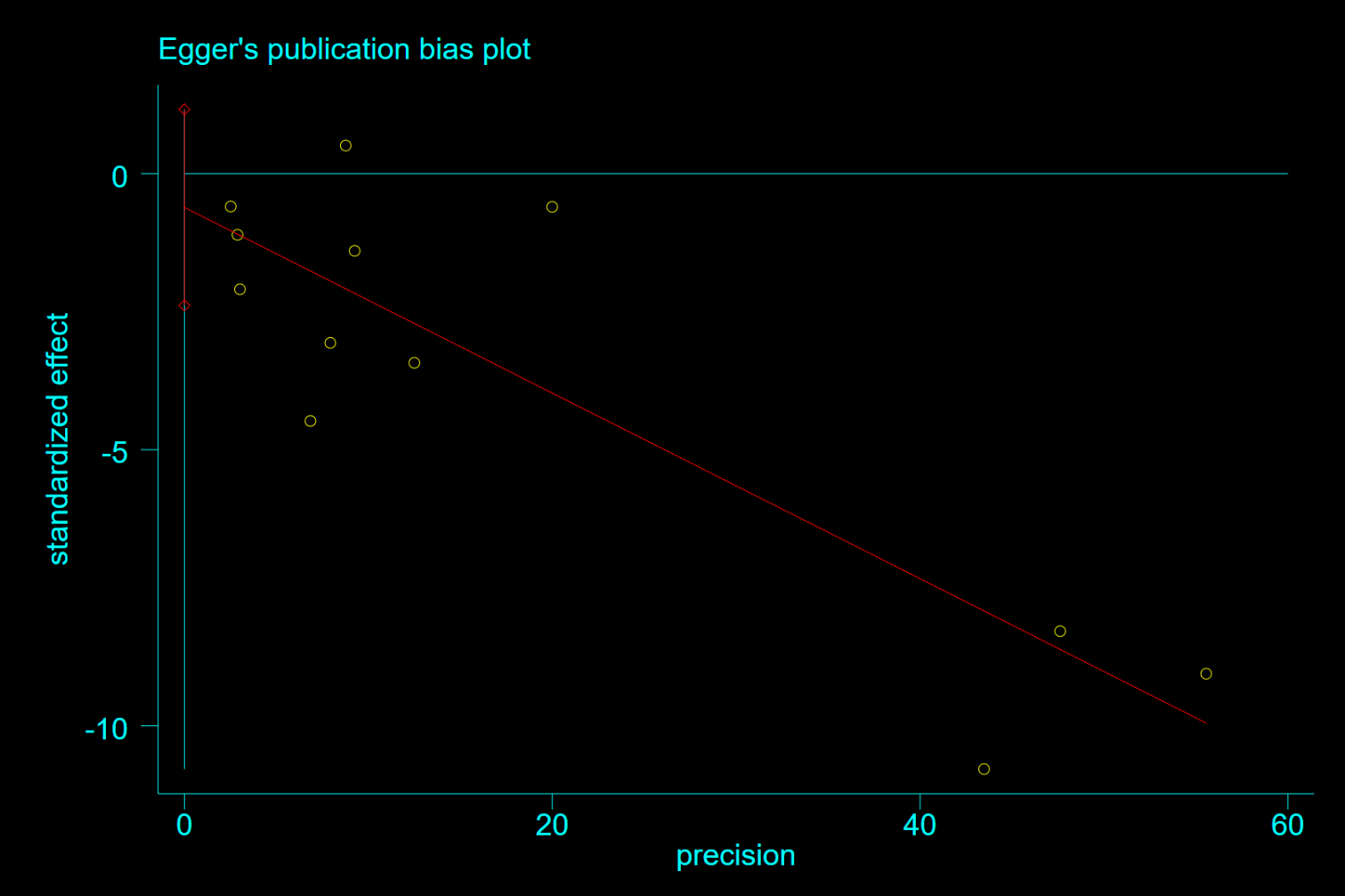


**Supplementary Figure 5**. Egger test(P=0.461) for hazard ratio of all-cause mortality among patients with metformin therapy vs. non-metformin therapy

Note: Begg test(P=0.373)


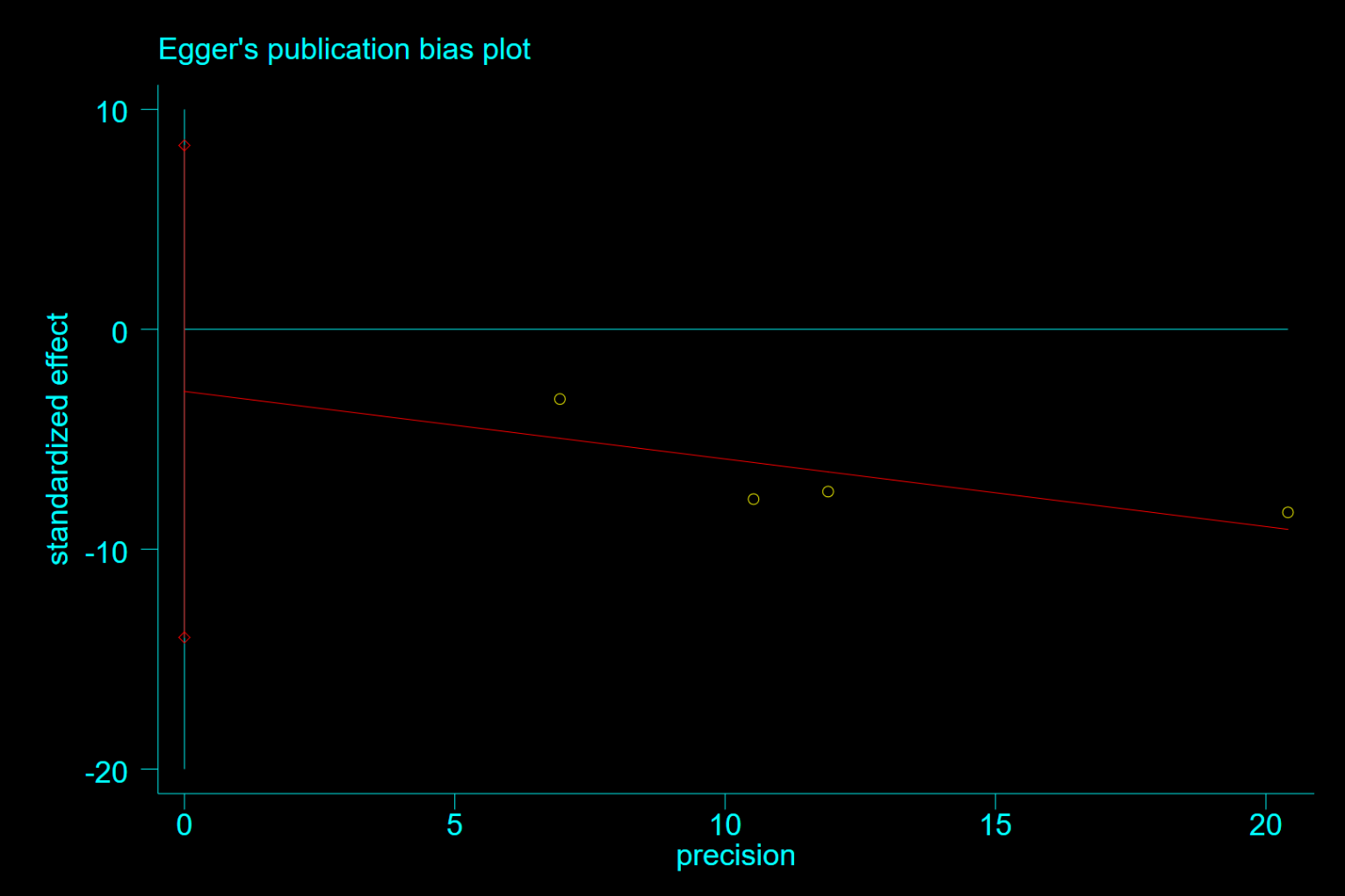


**Supplementary Figure 6**. Egger test(P=0.391) for hazard ratio of all-cause mortality among patients with metformin therapy vs. sulphonylurea therapy

Note: Begg test(P=0.734)


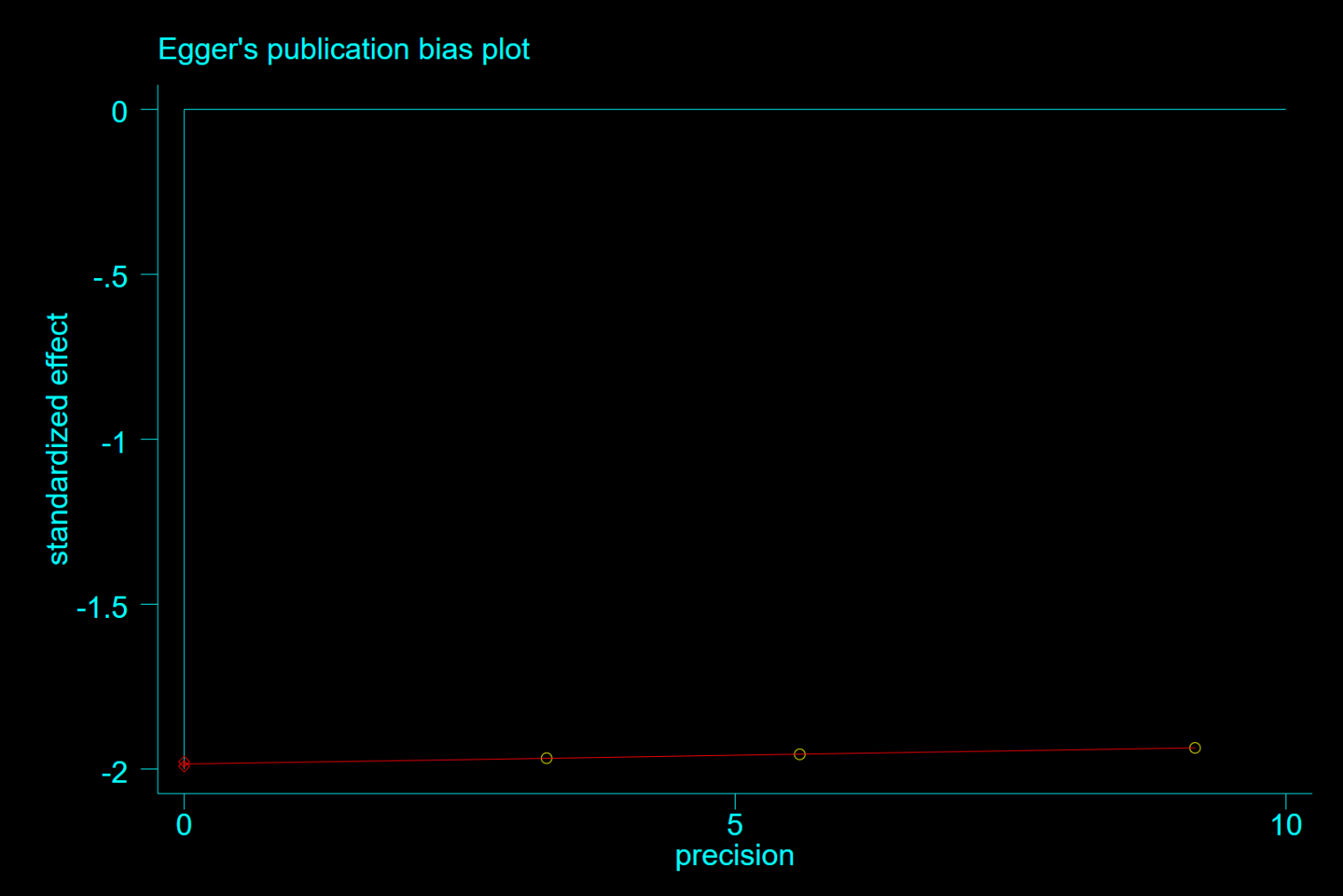


**Supplementary Figure 7**. Egger test(P=0.000) for hazard ratio of all-cause mortality among patients with metformin vs. diet therapy

Note: Begg test(P=0.296)


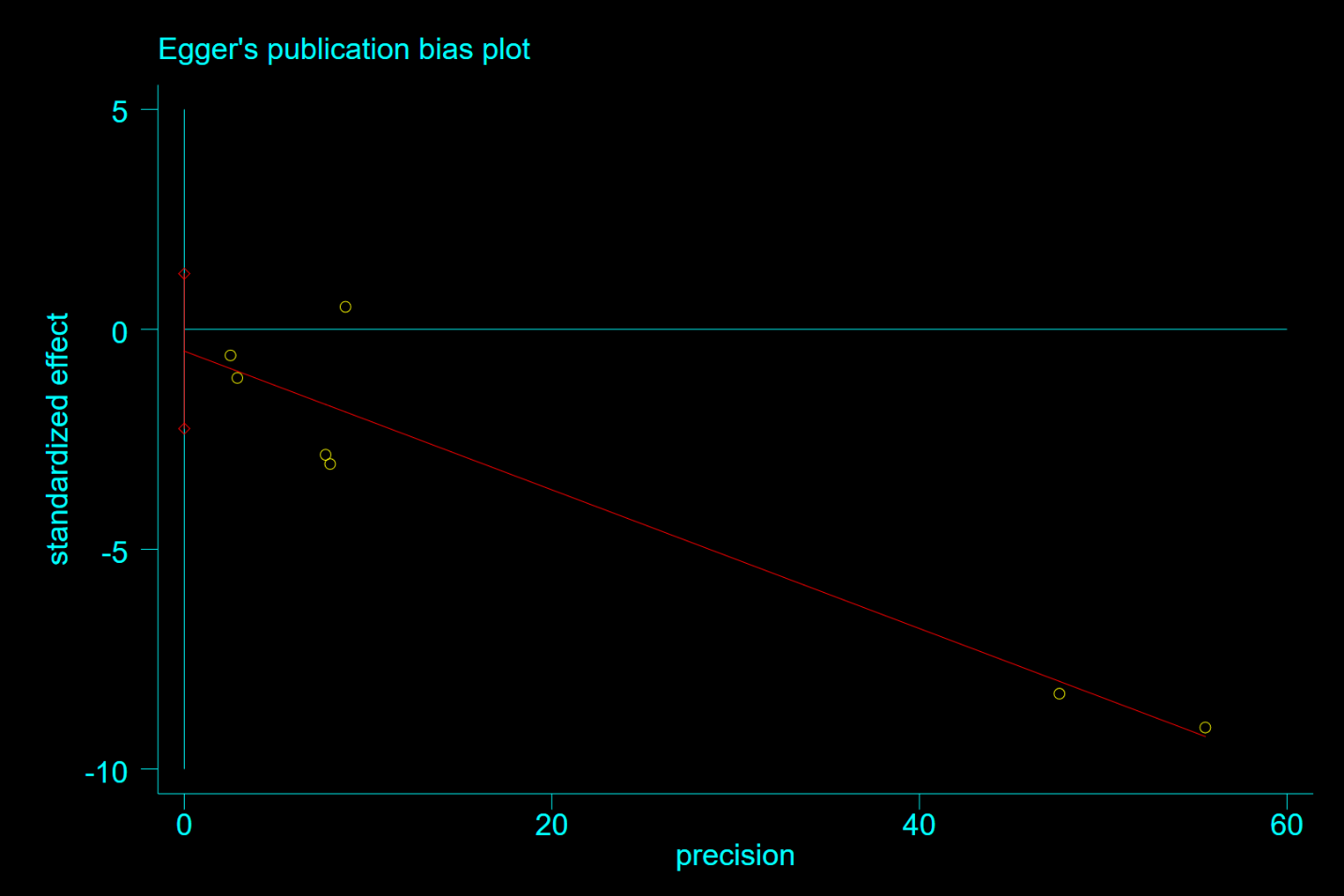


**Supplementary Figure 8**. Egger test(P=0.501) for hazard ratio of all-cause mortality among heart failure patients with metformin therapy vs non-metformin therapy

Note: Begg test(P=0.764)


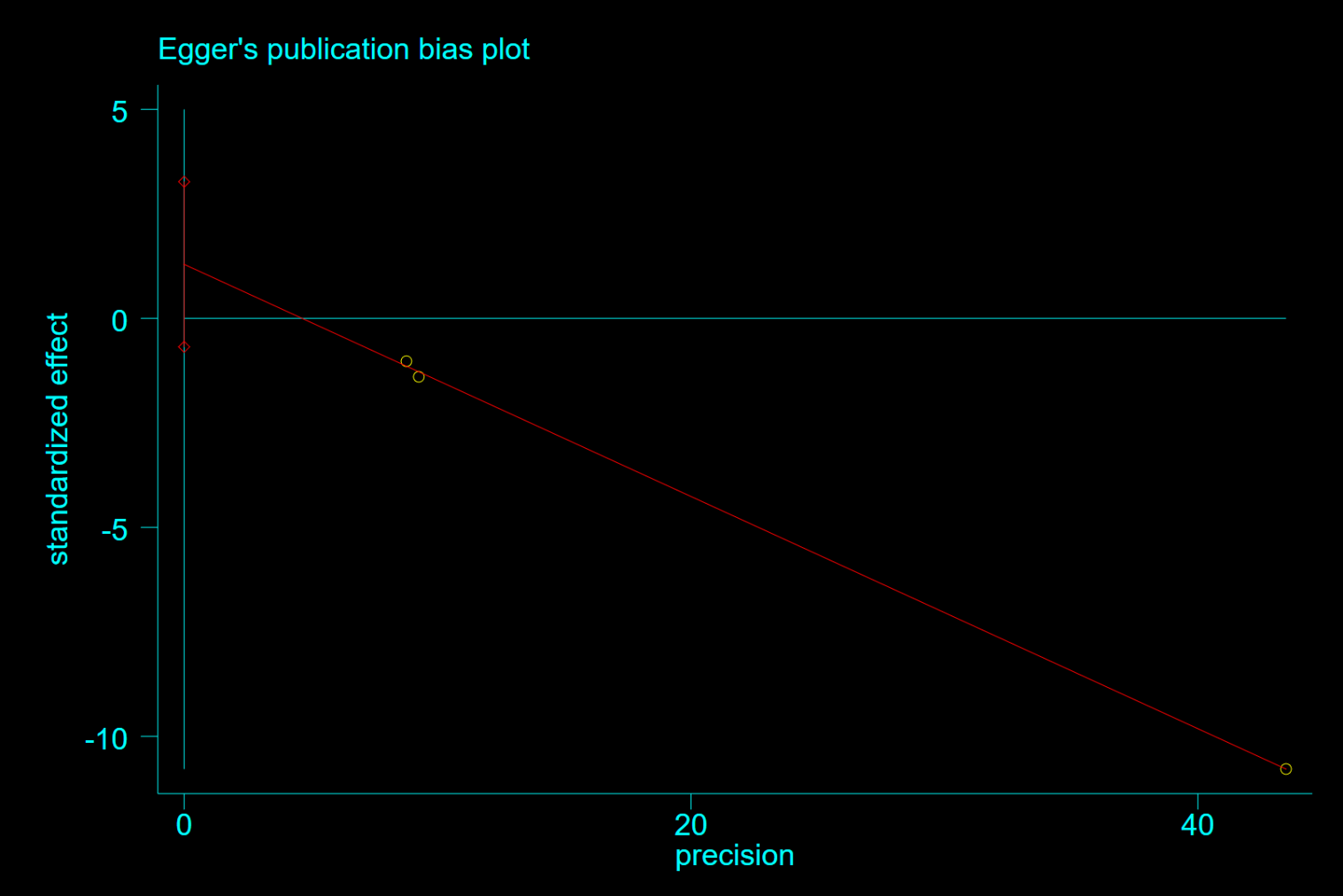


**Supplementary Figure 9**. Egger test(P=0.076) for hazard ratio of all-cause mortality among patients with CKD treated with metformin therapy vs non-metformin therapy

Note: Begg test(P=0.296)


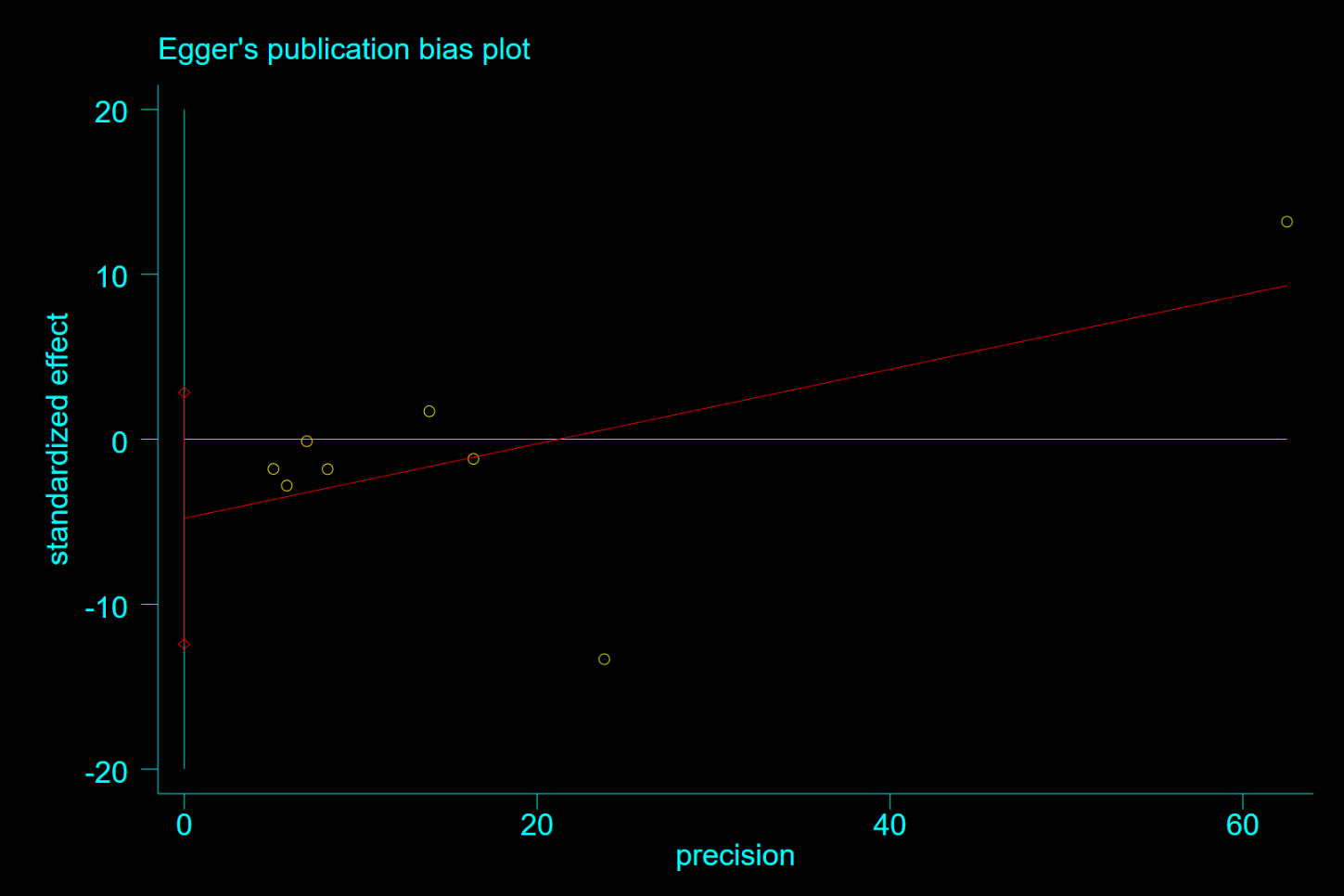


**Supplementary Figure 10**. Egger test(P=0.175) for hazard ratio of hospitalization among patients with metformin therapy vs non-metformin therapy

Note: Begg test(P=0.902)


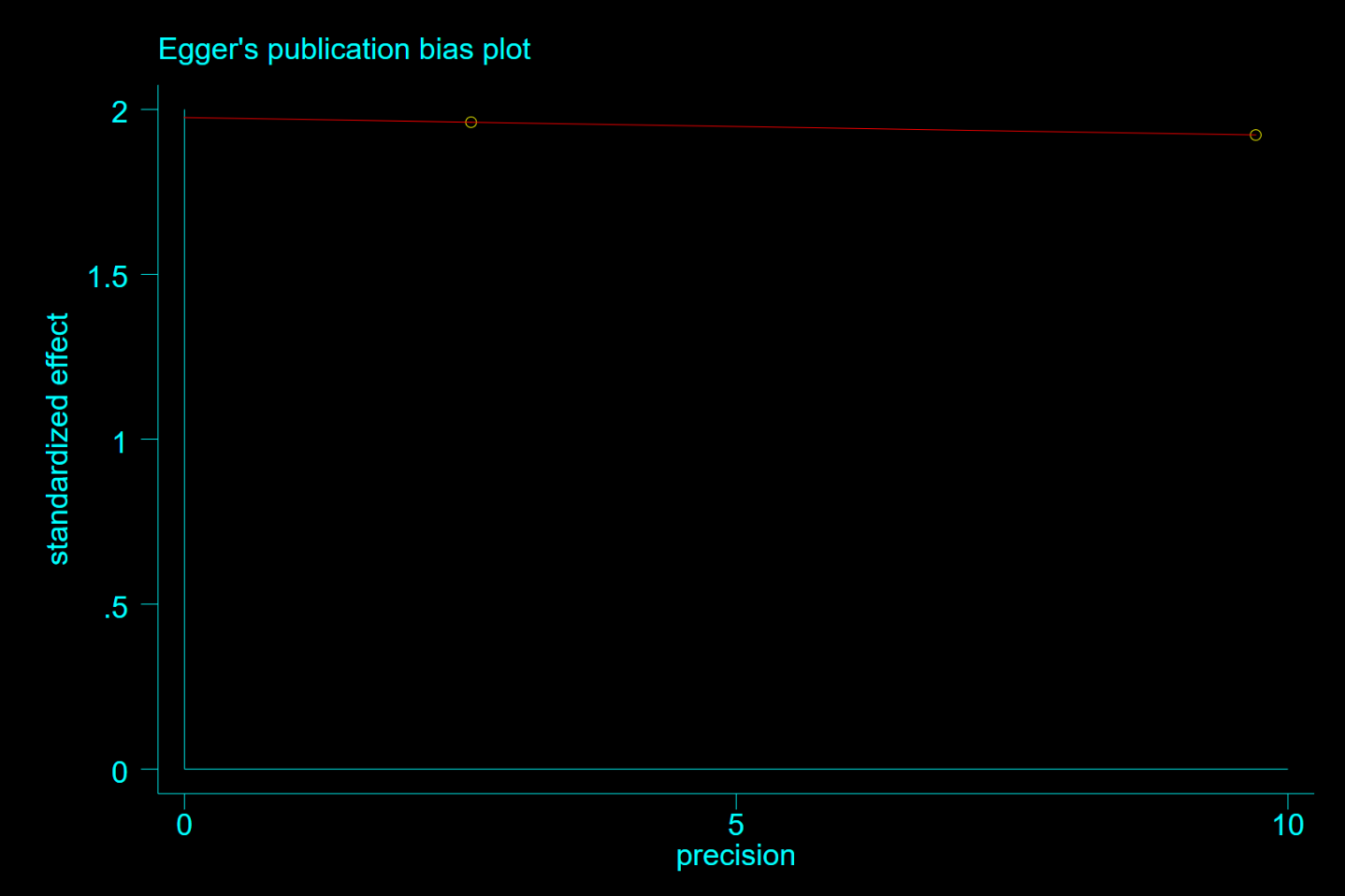


**Supplementary Figure 11.** Egger test for for hazard ratio of hospitalization among patients with metformin therapy vs SGLT-2i

Note: Begg test(P=1.0)


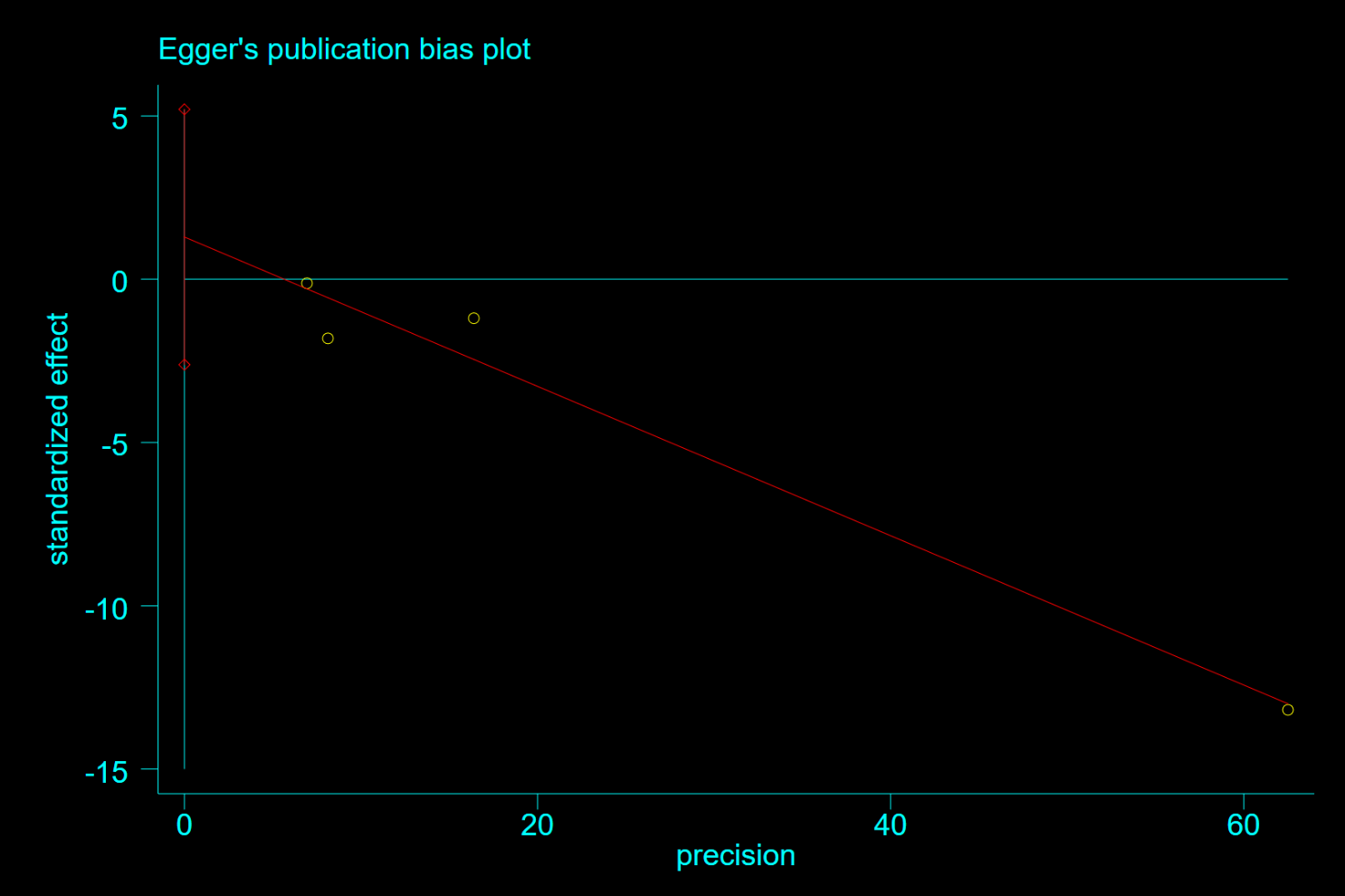


**Supplementary Figure 12**. Egger test(P=0.291) for hazard ratio of hospitalization among heart failure patients with metformin therapy vs non-metformin therapy

Note: Begg test(P=0.734)


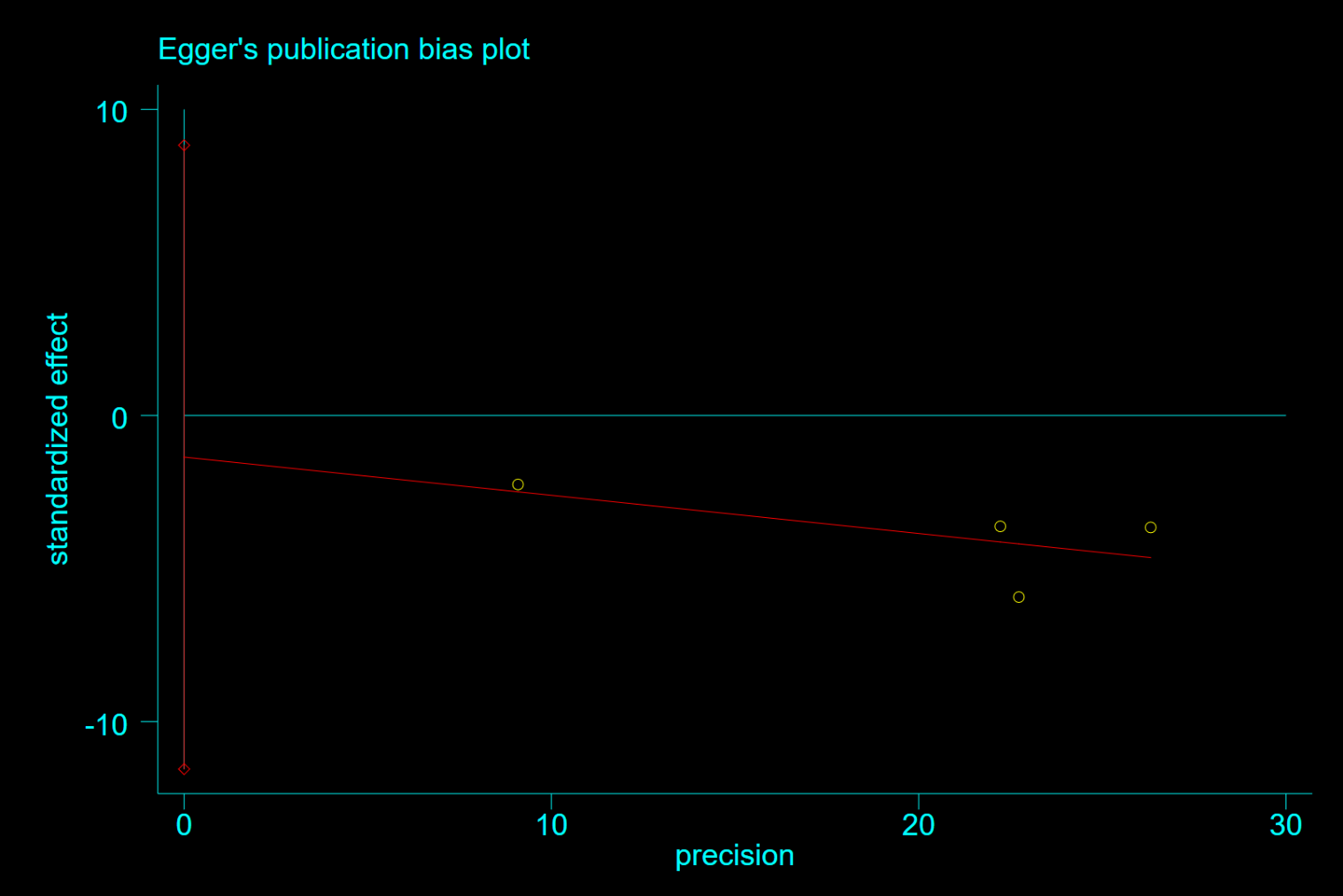


**Supplementary Figure 13**. Egger test(P=0.624) for hazard ratio of hospitalization among patients with metformin therapy vs sulphonylurea therapy

Note: Begg test(P=0.734)


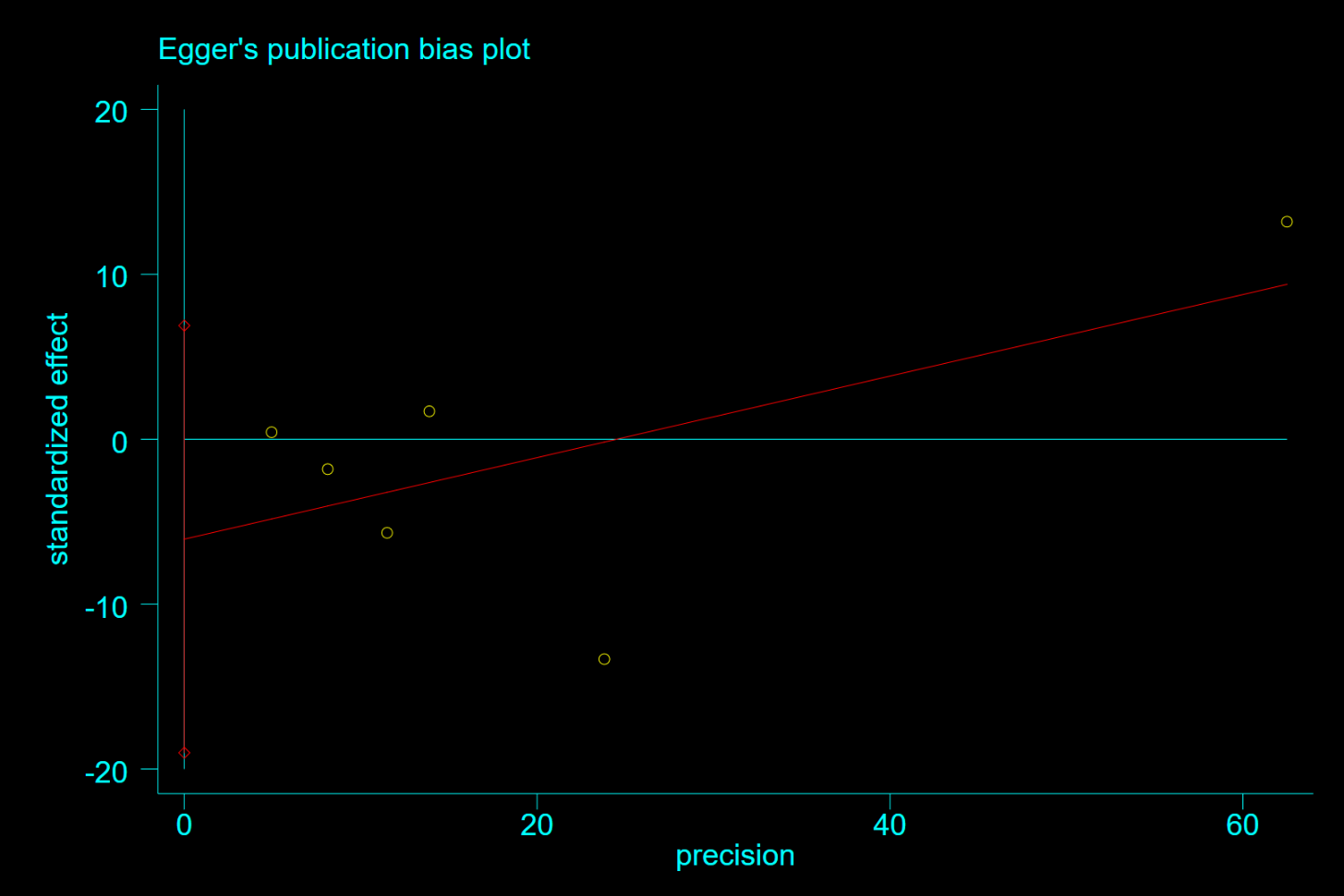


**Supplementary Figure 14**. Egger test(P=0.264) for hazard ratio of heart failure among patients with metformin therapy vs non-metformin therapy

Note: Begg test(P=1.0)


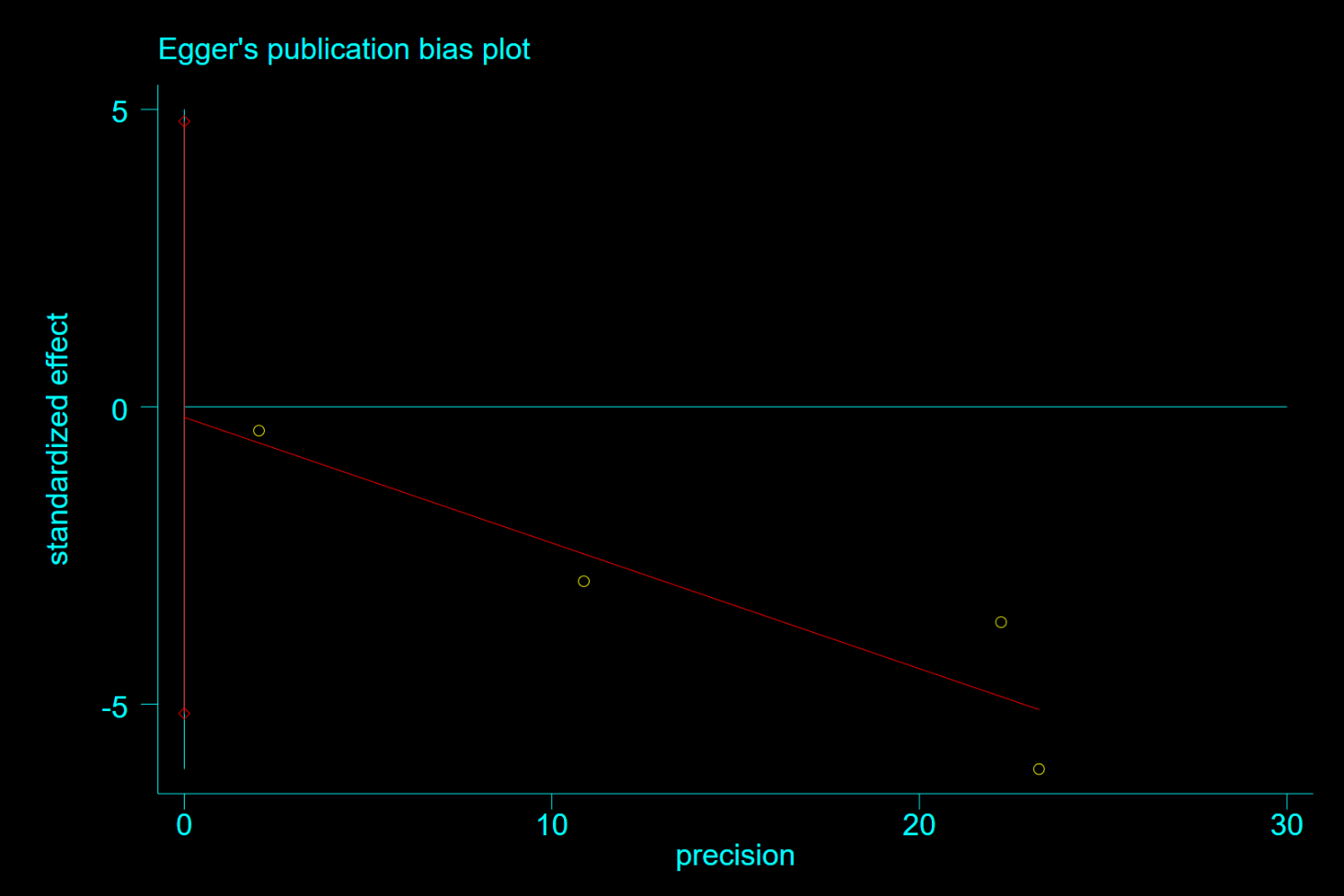


**Supplementary Figure 15**. Egger test(P=0.891) for hazard ratio of heart failure among patients with metformin therapy vs sulphonylurea therapy

Note: Begg test(P=0.734)


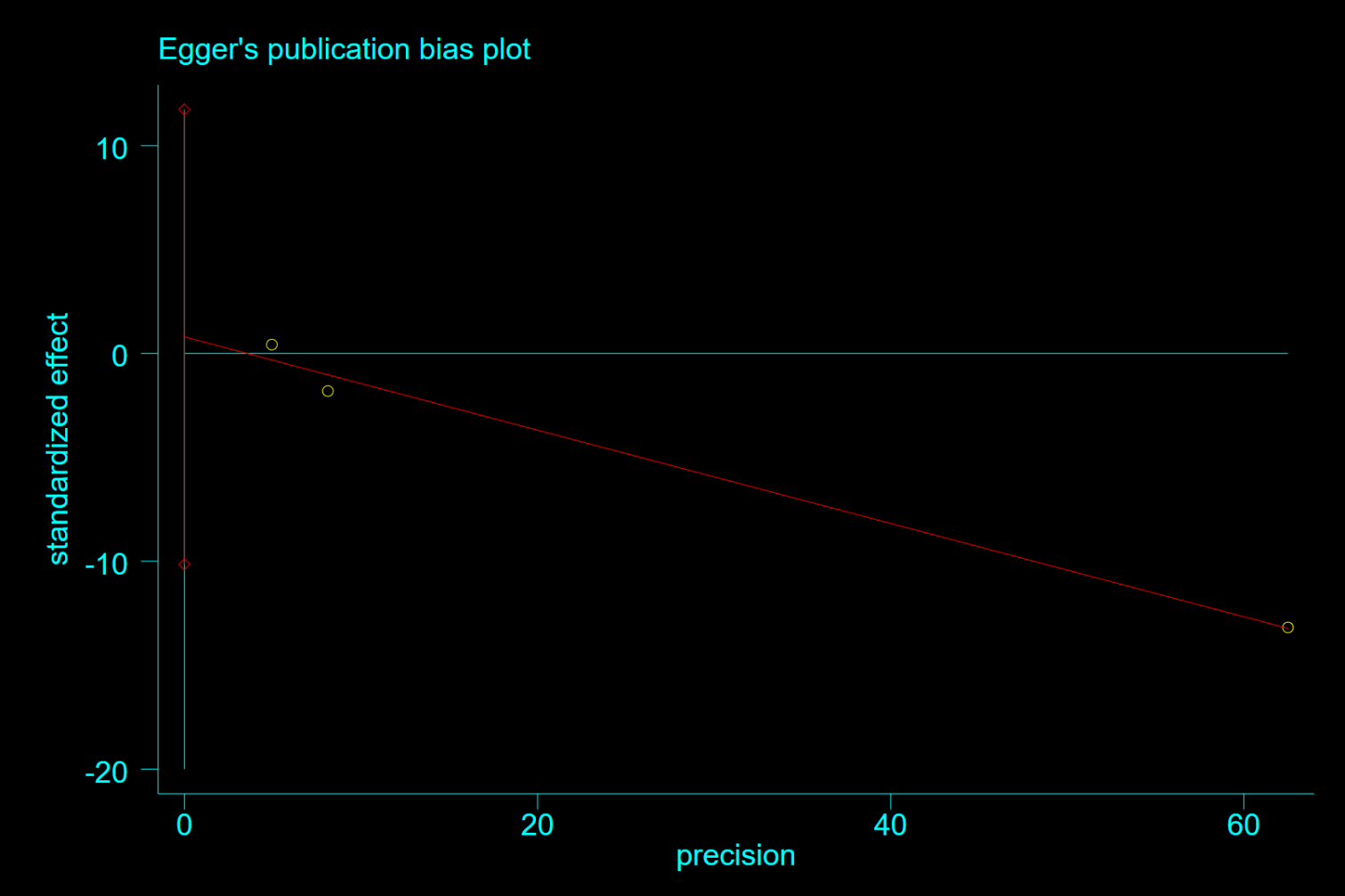


**Supplementary Figure 16**. Egger test(P=0.525) for hazard ratio of recurrent incident of heart failure among heart failure patients with metformin therapy vs non-metformin therapy

Note: Begg test(P=0.296)


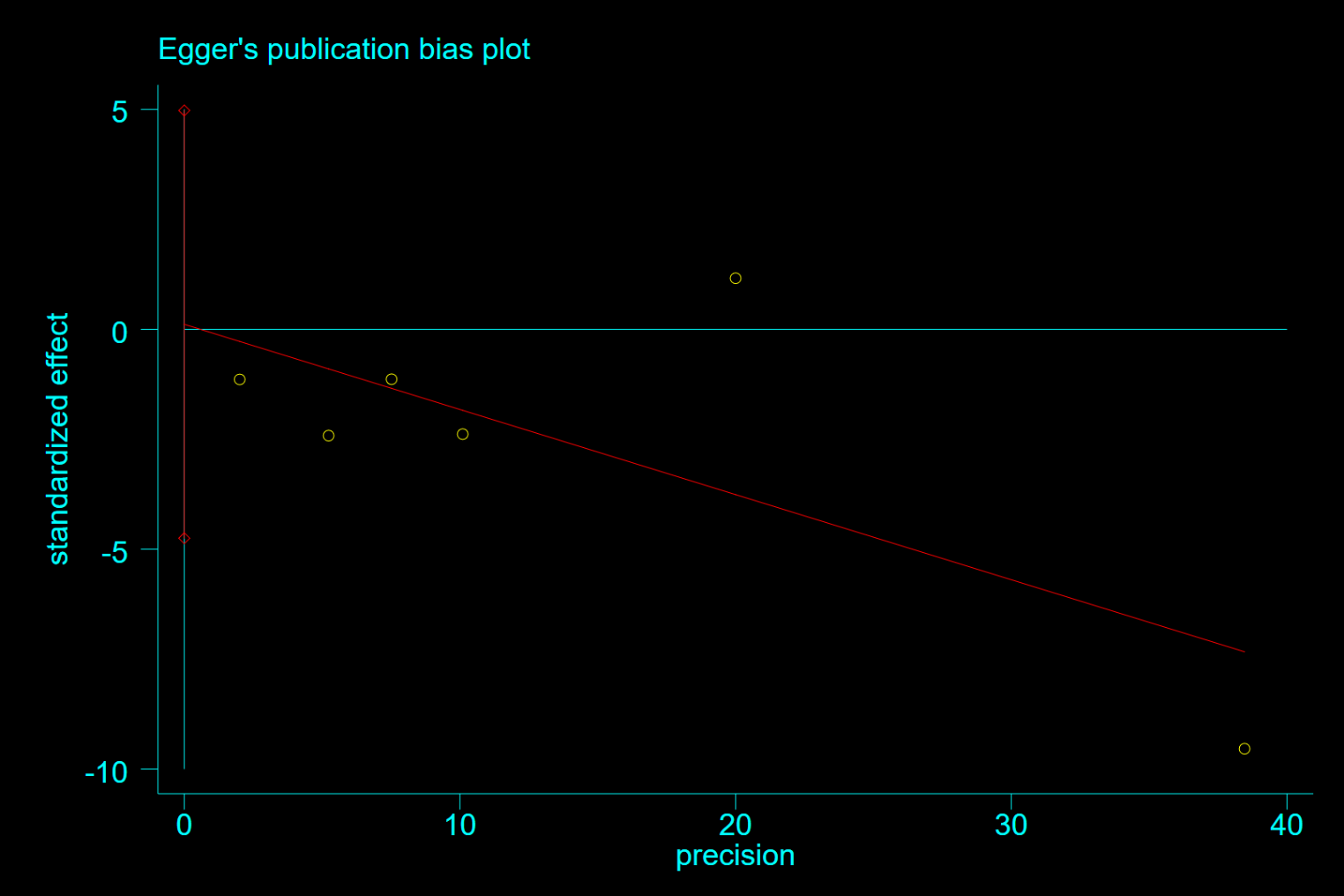


**Supplementary Figure 17**. Egger test(P=0.952) for hazard ratio of cardiovascular mortality among patients with metformin therapy vs non-metformin therapy

Note: Begg test(P=1.0)


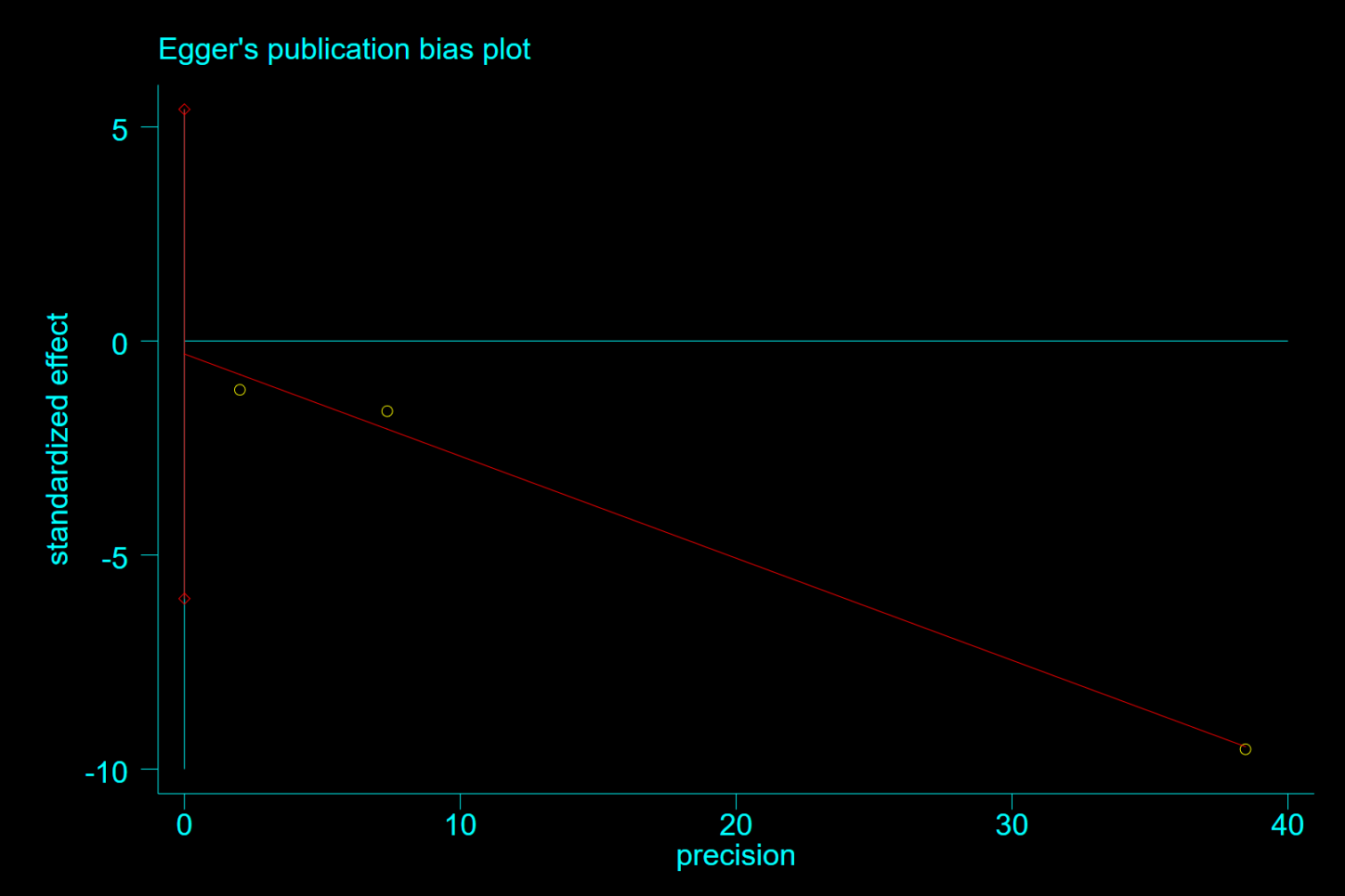


**Supplementary Figure 18**. Egger test(P=0.621) for hazard ratio of cardiovascular mortality among heart failure patients with metformin therapy vs non-metformin therapy

Note: Begg test(P=1.0)


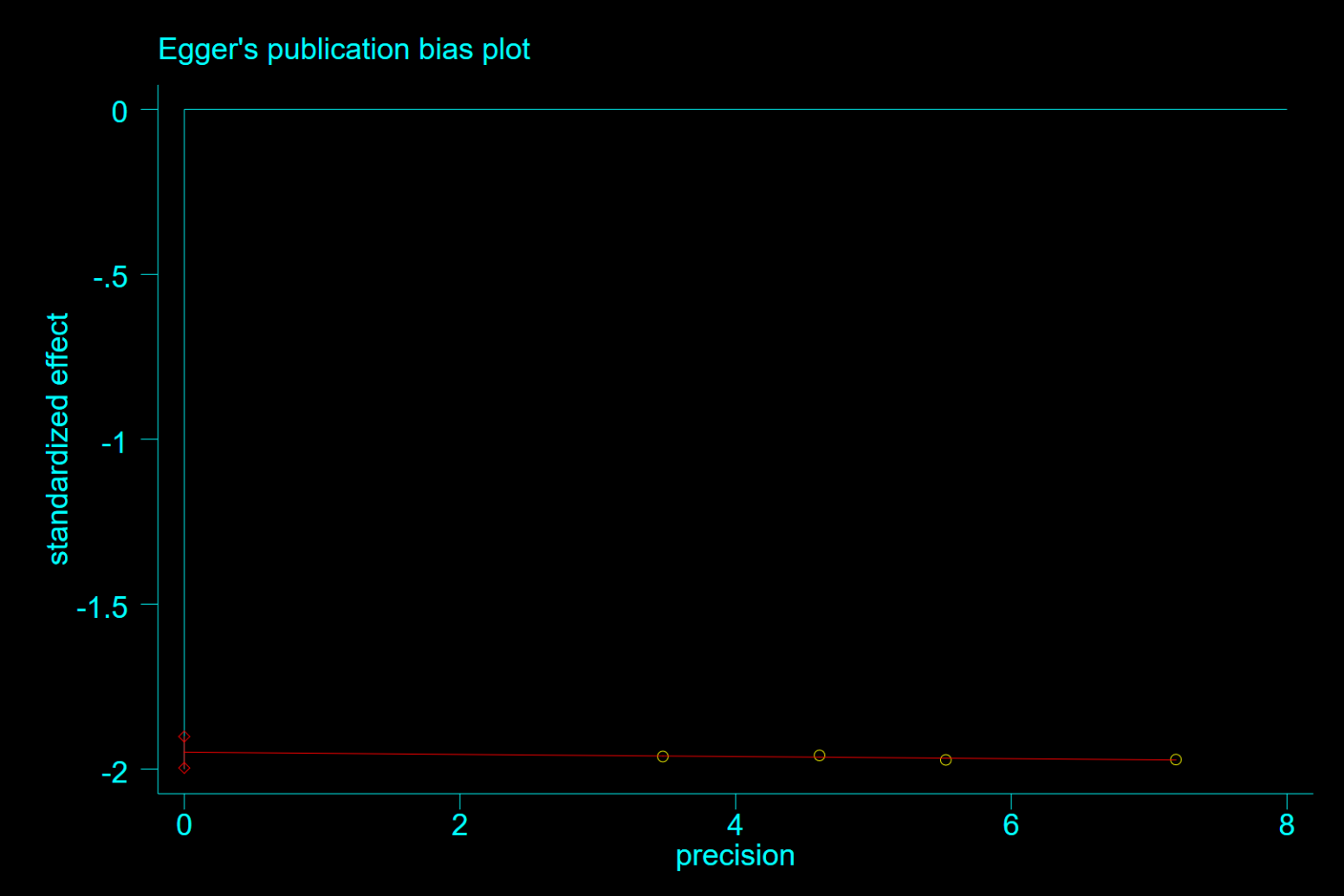


**Supplementary Figure 19**. Egger test(P=0.000) for hazard ratio of cardiovascular mortality among patients with metformin therapy vs sulphonylurea

Note: Begg test(P=0.089)


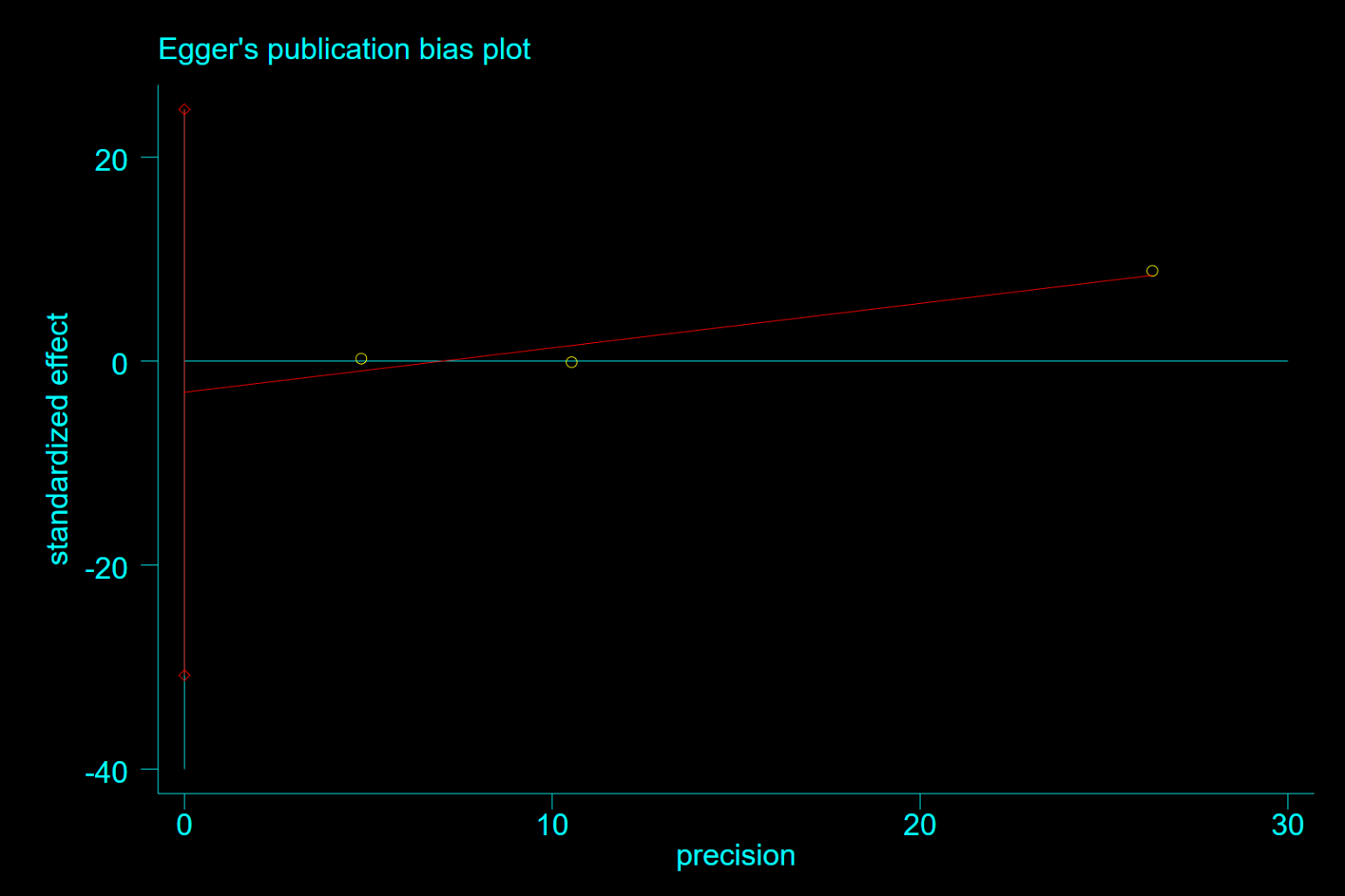


**Supplementary Figure 20**. Egger test(P=0.395) for for hazard ratio of stroke among patients with metformin therapy vs non-metformin therapy

Note: Begg test(P=1.0)


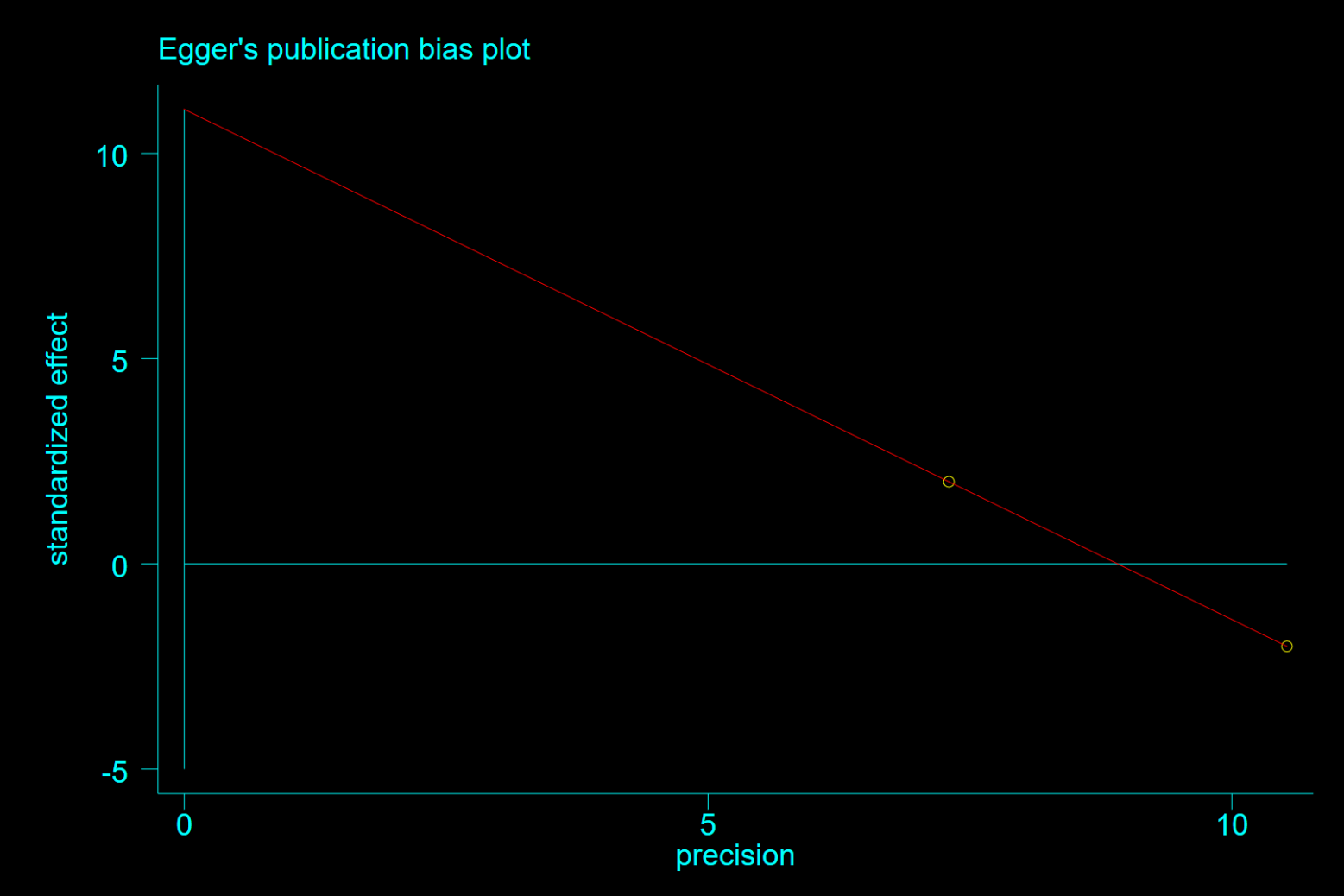


**Supplementary Figure 21.** Egger test for for hazard ratio of stroke among patients with metformin therapy vs SGLT2i

Note: Begg test(P=1.0)


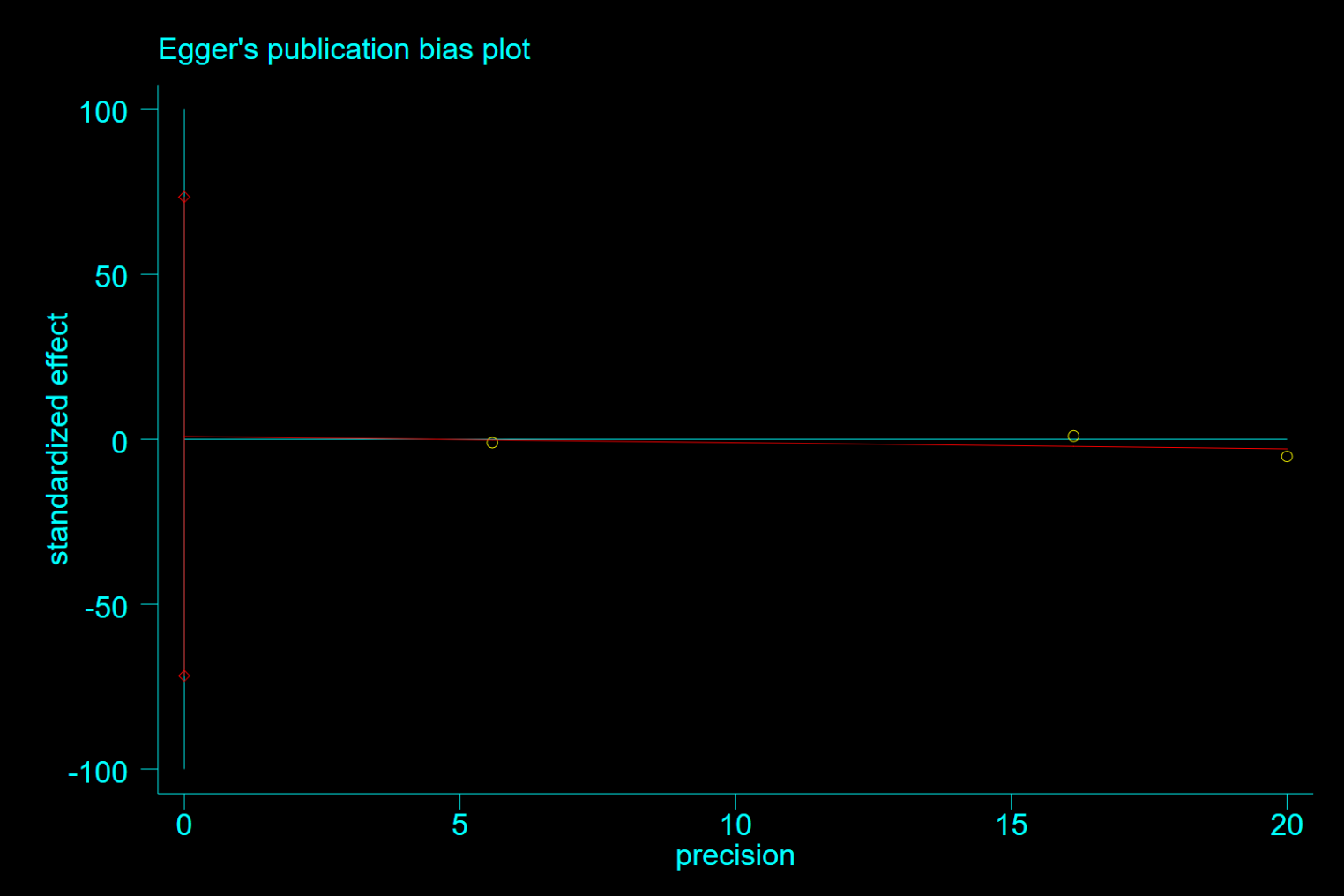


**Supplementary Figure 22**. Egger test(P=0.905) for for hazard ratio of AMI among patients with metformin therapy vs non-metformin therapy

Note: Begg test(P=1.0)
